# Multiomics Approach To Decipher the Origin of Chlorophyll Content in Virgin Olive Oil

**DOI:** 10.1021/acs.jafc.2c00031

**Published:** 2022-03-15

**Authors:** Carlos Quiles, Isabel Viera, María Roca

**Affiliations:** Group of Chemistry and Biochemistry of Pigments, Food Phytochemistry Department, Instituto de la Grasa, Consejo Superior de Investigaciones Científicas (CSIC), University Campus, Building 46, Carretera de Utrera km. 1, Sevilla 41013, Spain

**Keywords:** virgin olive oil, chlorophyll, green color, olive fruit, phyllobilins, pheophytinase, SGR

## Abstract

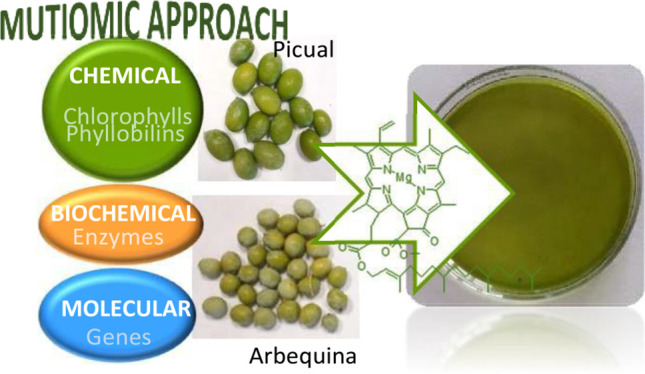

The color of virgin olive oils, ranging from intense green to brown-yellow, is one of the main selection factors for consumers and a quality criterion in specific legislations. Such coloration is due to their chlorophyll content and depends on the composition of the olive fruit. Through analytical chemistry (HPLC-hrMS^*n*^), biochemistry (enzymatic activity), and molecular biology (qRT-PCR) approaches, we have analyzed the origin of the differences in the chlorophyll content among several varieties of olive fruit throughout their ripening process. The higher chlorophyll biosynthetic capacity in olive fruits is due to the enzyme protochlorophyllide reductase, whereas chlorophyll degradation is accomplished through the stay-green and pheophytinase pathways. For the first time, the implication of chlorophyll dephytylase during the turnover of chlorophylls in fruit is shown to be responsible for the exclusive accumulation of dephytylated chlorophyll in Arbequina fruit. The multiomics results excluded the in vivo participation of chlorophyllase in chlorophyll degradation in olive fruits.

## 1 Introduction

Color is one of the fundamental factors that determine the consumer’s preference during food selection. It has been demonstrated that the color of virgin olive oil affects quality appreciation^1,2^ by the consumer. Its color varies from intense green to light yellow, and each market has its preference. Classically, eastern countries have preferred deep green olive oils, and this trend later became widespread throughout western countries as well. This tendency is associated with the new worldwide predilection toward early-harvested olive oils extracted exclusively from green olives, which are more flavorful and have a higher chlorophyll content. This current trend is in line with growing evidence of the health properties of chlorophyll as having antioxidant, antimutagenic, and chemopreventive properties, among others,^3^ leading to its presence becoming more valued in virgin olive oil. The chlorophyll composition of virgin olive oil depends on the chlorophyll content of the respective fruits (*Olea europaea* L.), which are solubilized during the extraction process.^4,5^ The chlorophyll content in the olive fruits varies greatly in the variety’s function and the ripening stage. There are olive varieties of high pigmentation (for example, Picual) and low pigmentation (such as Arbequina).^6^ The olive harvesting period coincides with the chlorophyll breakdown period. Olive fruits are green during the development phase; however, when the fruit initiates the ripening phase, chlorophyll degrades concomitantly with the synthesis of anthocyanins that will cover the fruit, turning it black. Consequently, green olive fruits, at the beginning of the harvest period (October), produce oil with higher chlorophyll content than the oil obtained from ripened olive fruits (December) at the end of the harvest.^4^

**Figure 1:**
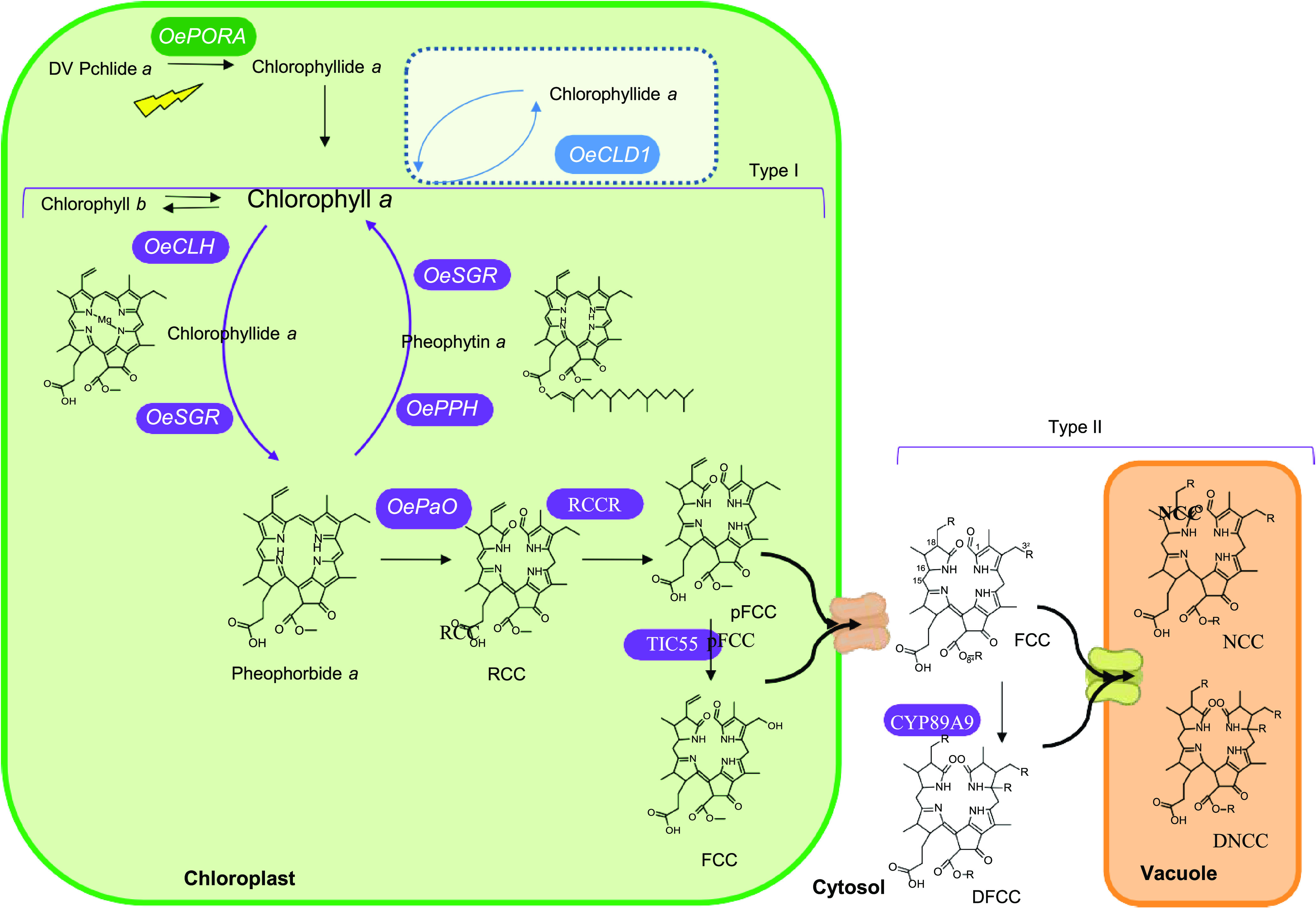
Schematic model of the chlorophyll metabolic pathway.

In any case, the chlorophyll content in the fruit, vegetable, or photosynthetic organism is a consequence of the balance between synthesis and degradation metabolism, with both pathways being developmentally programmed.^7^ In this sense, the biochemical reactions involved in the chlorophyll biosynthetic pathway are fully understood,^8^ and several regulators have been identified. Among all the biosynthetic enzymes, protochlorophyllide oxidoreductase (POR), which reduces protochlorophyllide at the C17-C18 double bond to form chlorophyllide (Figure 1), stands as a vital enzyme^9^ responsible for the regulation of the route, interacting with up- and downstream enzymes and regulators. Recent findings have revealed an additional essential role of POR in orchestrating the synergy between chlorophyll synthesis and the photosynthetic membrane biogenesis in plants.^10^ During senescence (or ripening), chlorophyll breaks down. Although the biochemical pathway has been roughly deciphered, several questions remain. Classically, it has been assumed that chlorophyll is successively degraded by chlorophyllase (CHL), which eliminates phytol, followed by a substance-chelating metal that substitutes central magnesium with hydrogen, ultimately creating pheophorbide. However, it has been shown, at least in senescent leaves, that these reactions were catalyzed in the reverse order by alternative enzymes^11^ (Figure 1). First, chlorophyll is dechelated by stay-green (SGR), forming pheophytin,^12^ which is next dephytylated by pheophytinase (PPH). However, the implication of the new alternative enzymatic system (SGR/PPH) in ripened fruits is unclear,^13^ with an assumption that chlorophyll dephytylation in fruits is indistinctly attributed to CHL^14^ or PPH.^15^ Independent of the chlorophyll pathway, the chlorophyll degradation pathway continues from pheophorbide *a*, next being oxygenolytically opened by pheophorbide *a* oxygenase (PaO) and forming the first linear chlorophyll catabolites. The groups of linear chlorophyll catabolites are globally termed as “phyllobilins.”^16^ This first catabolite is named red chlorophyll catabolite (RCC) and is channeled to RCC reductase (RCCR) to create the fluorescent chlorophyll catabolite (FCC). FCCs are exported to the cytosol, where they are modified and then imported into the vacuole and tautomerized nonenzymatically to the final chlorophyll catabolites, which are nonfluorescent chlorophyll catabolites (NCCs) and dioxobilin-type NCCs (DNCCs) (Figure 1).^16,17^ All the phyllobilins share a common skeleton, and more than 40 different structures have been identified.^12^ The differences between individual phyllobilins are mainly restricted to three positions (although new substitutions have been described recently) with constrained functional groups (Figure 1). At C-18, the vinyl group could be dihydroxylated; at C-3^2^, esterification is possible with the O-β-glycopyranosyl, O-β-malonyl, or O-β-(6′-O-malonyl) glucopyranosyl groups; and finally, the methyl ester function at C-8^2^ can be hydrolyzed or not.

This chlorophyll degradation pathway is responsible for the massive and net degradation of chlorophyll (and the consequent loss of green color) that occurs during leaf senescence or fruit ripening (PaO or phyllobilin pathway).^16^ Additionally, the chlorophyll present in photosynthetic tissues is subjected to continuous turnover, with a shelf-life estimated at several hours.^18^ This means chlorophyll is, independent of the developmental stage, continuously synthesized and degraded, maintaining a steady state, without a net increase or decrease in the chlorophyll concentration, coupled to the photosystem II (PSII) repair system. However, the reactions implied in the synthesis and degradation during turnover are largely unknown. At present, only chlorophyll dephytylase (CLD1, Figure 1) has been identified as the enzyme that catalyzes the de-esterification of phytol during chlorophyll turnover^19^ in *Arabidopsis*. Furthermore, chlorophyll can also be the substrate of oxidative metabolism, most likely through peroxidases, forming metabolites such as 13^2^-hydroxy-chlorophyll and 15^1^-hydroxy-lactone-chlorophyll.^20^ At present, however, the physiological relevance of the oxidative reactions is uncertain because it is unknown how they are further degraded and how they are integrated into the phyllobilin pathway.

Chlorophyll metabolism in olive fruits has been investigated, mainly by analyzing the evolution of the colored chlorophyll metabolites through the development and ripening of the olive fruit^4^ and the multiple varieties,^6,21^ focusing on the quantification of dephytylated chlorophyll (chlorophyllide and pheophorbide). This analytical methodology has been combined in certain studies with enzymatic determinations, specifically with the role of chlorophyllase as the first catabolic enzyme.^4,6^ However, recent advances in our understanding of chlorophyll metabolism (at the analytical chemistry, biochemical, and genetic levels) open new lines of research to investigate in depth the chlorophyll metabolism in olive fruits as the origin of virgin olive oil color. As stated previously, the green color is a highly appreciated attribute in virgin olive oils. High-quality extra virgin olive oils are an intense shade of green, whereas low-quality, refined, or storage olive oils are more yellow. For example, one of the “fraudulent” practices in the virgin olive oil market is the addition of green colorants to cheaper olive oils to sell them as high-priced green virgin olive oils.^22^ Undoubtedly, the association between green color with “freshness” and the increasing trend of early harvesting is moving the market even more toward green virgin olive oils. Consequently, deciphering the mechanisms responsible for the chlorophyll content in olive fruits becomes essential in controlling olive oil’s green color. With that aim, we have applied for the first time a holistic approach, taking into account analytical chemistry, biochemistry, and molecular factors, to study the reasons for the chlorophyll content in olive fruits.

## 2 Materials and Methods

### 2.1 Plant Material

The study was carried out with olive fruits (*Olea europaea* L.) of Picual and Arbequina varieties from the experimental farm of the Andalusian Institute of Agricultural and Fisheries Research and Training (IFAPA), Centro Alameda del Obispo of Córdoba (Spain). A total of 2 kg of fruits were collected from six olive trees randomly chosen every month from September to December. The olive fruits were taken from the branches that were as high as the extended arms, throughout all the perimeter of the tree, and from the outer and inner areas. Pigment extraction and enzymatic activities were determined in fresh fruit within 1 week, while gene expression was measured with frozen and ground exocarp and mesocarp in liquid nitrogen and stored at −80 °C. For PaO-RCCR enzymatic determinations, pepper fruits in a single advanced maturity stage (red color) were acquired in a supermarket. Arabidopsis seeds for PPH activity (Col-0, European Arabidopsis Stock Centre, Nottingham, UK) and spinach seeds for NCC standard extraction were sowed in vermiculite. After germination, green leaves were collected and induced to senescence under dark conditions in Petri dishes on moistened filter paper discs during 5–7 days at 25 °C until the prevailing coloration was yellow.^23^

### 2.2 Extraction, Separation, and Quantification of Chlorophyll

For each analysis, samples of 4 to 15 g (depending on the degree of ripeness of the fruits) were taken from 100 destoned fruits homogenized. Pigment extraction was performed with *N*,*N*-dimethylformamide, and the chlorophyll fraction was purified from the lipids and carotenes by successive liquid extractions with hexane.^6^ All analyses were performed in triplicate under green light. The pigments were separated by reverse-phase high-performance liquid chromatography (HPLC) using a Hewlett-Packard HP 1100 liquid chromatograph. A Mediterranea Sea18 column (20 cm × 0.46 cm, 3 μm) was used (Teknokroma, Barcelona, Spain) protected by a guard column (1 cm × 0.46 cm) packed with the same material. Separation was performed using an elution gradient^6^ with the mobile phases: water/1 M ammonium acetate/methanol (1/1/8, v/v/v) and methanol/acetone (1/1, v/v). The online ultraviolet–visible (UV–vis) spectra were recorded from 350 to 800 nm with a photodiode array detector and sequential detection at 410, 430, 450, and 666 nm. Data were collected and processed with an LC HP ChemStation (Rev.A.05.04). Identification of chlorophyll derivatives was made by co-chromatography with authentic samples and from their spectral characteristics previously identified^24^ by electrospray ionization (ESI)/APCI-*hr*-HPLC–mass spectrometry (MS)^2^. Quantification of pigments was performed with the corresponding calibration curves (amount versus integrated peak area). The calibration equations were obtained by the least-squares linear regression analysis over a concentration range according to the observed levels of these pigments in the analyzed samples. For each standard solution, duplicate injections were made for five different volumes.

### 2.3 Extraction, Separation, and Identification of Phyllobilins

Fresh tissue was extracted with potassium phosphate buffer pH 7.0/methanol (1:3, v/v), centrifuged, and concentrated using an SPE column (Bakerbond C18 SPE, 500 mg/6 mL, J.T. Baker, Deventer Holland). Phyllobilins were analyzed with a Dionex Ultimate 3000RS U-HPLC (Thermo Fisher Scientific, Waltham, MA, USA) using a C18 HPLC column of 3 μm (Spherisorb ODS-2, Teknokroma, Barcelona, Spain) (20 × 0.46 cm i.d.). The gradient has been previously published;^25^ the eluent 0.1% formic acid was mixed in water and methanol, with a flow rate of 1 mL/min. The MS system was a microTOF-QII high-resolution time-of-flight mass spectrometer (UHR-TOF) with a qQ-TOF geometry (Bruker Daltonics, Bremen, Germany) equipped with an ESI interface. The method scans from *m/z* 50 to 1200 at positive mode and the mass spectra were registered in MS full scan mode. MS^2^ analysis was also acquired in Auto-MS/MS for deeper characterization. Bruker Daltonics TargetAnalysis 1.2 software allowed for the identification of a specific compound in the base to mass accuracy and the isotopic pattern. Both parameters were determined using the SigmaFit algorithm, establishing their maximum limits at 5 ppm and 50.^25^ We have developed a phyllobilin library for screening, which includes monoisotopic masses, elemental composition, retention time, and characteristic fragment ions for more than 40 phyllobilins already identified.^24^ Additionally, the SmartFormula3D software was also utilized for the study of the MS^2^ data.

### 2.4 Determination of Chlorophyllase Activity

First, a protein concentrate was obtained through a complete pulverization and repeated rinsing of tissue powder in cold acetone until a protein precipitate is obtained. Next, 0.5 g of acetone powder was extracted with 15 mL of 5 mM sodium phosphate buffer pH 7 containing 50 mM KCl and 0.24% Triton X-100. The supernatant was used as the crude extract after centrifugation. The reaction mixture contained 0.1 μmol chlorophyll *a* in acetone, 100 mM Tris buffer pH 8.5 containing 0.24% Triton X-100, and the enzyme extract in a 1:5:5 ratio. The enzymatic reaction run over 2 h at 50 °C, and the levels of chlorophyllide were quantified by HPLC, as described previously.^6^

### 2.5 Determination of PPH Activity

The extraction protocol was described previously,^11^ but the reaction had to be adapted to the olive fruit characteristics. Specifically, the reaction mixture contained 20 μL of pheophytin *a* (1 mM), 170 μL of 25 mM Tris-Mes pH 8.0 containing 5 mM l-ascorbic acid, and 50 μL of enzymatic extract. The enzymatic reaction run over 4 h at 40 °C, and the levels of pheophorbide *a* were quantified by HPLC, as described previously. To avoid the nonenzymatic formation of pheophorbide, it was necessary to include two blanks for each enzymatic determination, one at time zero and one reaction without the enzyme.

### 2.6 Determination of PaO/RCCR Activity

Approximately 2 g of fresh tissue (pepper and olive fruit) was homogenized with the extraction buffer (0.4 M sorbitol, 25 mM Tricine–KOH pH 8, 2 mM Na–EDTA, 1 mM MgCl_2_, 0.1% BSA, 5 mM PEG 4000, and 10 mM cysteamine–HCl) and span. Next, pellets were dissolved in the incubation buffer (extraction buffer without BSA), spun, and finally frozen at −80 °C. The thylakoid membranes were dissolved in buffer Tris-Mes pH 8 and spun. The soluble proteins included in the supernatant from pepper constitute the source of RCCR. The pellets from olive fruits were washed several times with buffer Tris-Mes pH 8, spun, and finally dissolved in buffer Tris-Mes pH 8 with 1% Triton X-100 for shaking for 30 min at 4°. Aliquots of the supernatant (proteins associated with membranes) are the source of PaO. The enzymatic assay contained the RCCR fraction (from pepper), PaO fraction (from olive fruits), cofactors (glucose-6-P, NADPH, glucose-6-P dehydrogenase, ferredoxin, and ferredoxin-NADPH-oxidoreductase), and pheophorbide a in Tris-Mes buffer, as described before.^23^ The activity was measured in the base to the formation of pFCCs for 1 h. The separation was developed through HPLC in the reverse phase with the same column as for chlorophyll quantification but with an isocratic elution gradient: 20% A (potassium phosphate buffer 50 mM pH 7)–80% B (deionized water/Pi-K buffer 50 mM pH 7/methanol at 1:1:8). FCCs were identified with a fluorescence detector (320/450 nm), retention times, and co-chromatography with authentic standards and quantified as fluorescence units (FU).^27^

### 2.7 Total RNA Extraction and cDNA Synthesis

Total RNA was extracted from 100 mg of collected tissue using the RNeasy Plant Mini Kit (Qiagen, Hilden, Germany), following the instructions of the manufacturer. Samples were then treated with RNase-free DNase set (Qiagen, Hilden, Germany) and purified and concentrated with RNA CLEAN & CONCENTRATOR-5 kit (Zymo Research, Irvine, USA), following manufacturer’s instructions. The RNA concentration was measured by the NanoDrop ONE C spectrophotometer, and the quality of the nucleic acids was checked on the agarose gel. For cDNA synthesis, one microgram of purified DNase-treated total RNA was used to prepare cDNA by reverse transcription with M-MuLV reverse transcriptase (New England BioLabs, Ipswich, USA) and oligo(dT)_18_ primer, according to the manufacturer’s protocol.

### 2.8 Gene Expression Analysis

Quantitative real-time polymerase chain reaction (PCR) was performed on a CFX96 C1000 Touch real-time PCR System (Bio-Rad, Milan, Italy) using the SsoAdvanced (2X) kit (Bio-Rad, Milan, Italy), according to manufacturer’s instructions and gene-specific primers. All reactions were performed twice in 96-well reaction plates, and negative controls were set. The amplification reactions were prepared in a final volume of 20 μL by adding 10 μL of the SsoAdvanced (2X) kit, 0.4 μL (10 μM) of each primer, and 5 μL (5 ng) of cDNA. The cycling parameters were as follows: one cycle at 95 °C for 3 min to activate the enzyme, followed by 40 cycles of denaturation at 95 °C for 10 s and annealing–extension at 60 °C for 30 s. To confirm the existence of a unique PCR product, the melting curve was evaluated by an increase of 0.5 °C every 5 s within the 65 to 95 °C range. A unique melting peak in every reaction was observed. The primer efficiency was determined by measuring a standard curve for each gene with four dilution points. The housekeeping genes, *18S*, GenBank accession number L49289.1; *GAPDH*, GenBank accession number 154260889; and *UBQ2*, GenBank accession number AF429430, were used to normalize the expression levels. The oligonucleotide primer sets used for Q-RT-PCR analysis (Table S1) were designed using Primer3 (http://bioinfo.ut.ee/primer3-0.4.0/).

### 2.9 Statistical Analysis

Pigment analysis and enzymatic determinations were carried out in triplicate, and data were expressed as means ± SD. The data were analyzed for differences among means using one-way analysis of variance (ANOVA). Tukey’s multiple-range test was used as a post hoc comparison of statistical significance (*p* < 0.05). The statistical studies were carried out with OriginPro 2020b software. To determine whether differences were statistically significant among gene expression analysis, *t*-tests were performed on five biological replicates for every ripening stage.

## 3 Results

### 3.1 Evolution of Chlorophyll Metabolites

The focus of this research concerned the analysis of fully developed fruits, which means the mesocarp and endocarp have reached their final weight, and no growing interferences are involved. However, the developmental stages analyzed included the end of the net chlorophyll synthesis period (September in Table 1), followed by the progressive net chlorophyll degradation period (October to December, Table 1). The ripening stages of the olive fruits analyzed in the present study were selected to coincide with the harvesting period for virgin olive oil extraction. As expected, the total chlorophyll content in fruits of the Picual variety was higher than that in Arbequina fruits, and dephytylated chlorophyll metabolites (chlorophyllide and pheophorbide *a*)^6^ were present exclusively in Arbequina fruits. Pheophytin *a* was present in both varieties, and its content decreased throughout the ripening process. As expected, only chlorophyll catabolites from the *a* series were detected, following the chlorophyll breakdown pathway (Figure 1). The presence of 13^2^-hydroxy-chlorophylls, mainly in Arbequina fruits, has been attributed to the activity of peroxidase in olive fruits,^20^ although its implication in the chlorophyll degradation pathway has been questioned.^12^ It is important to highlight that in both varieties, the higher breakdown of chlorophyll coincides with the synthesis of anthocyanins. However, this phenomenon occurs earlier in fruits of the Picual variety (November) than in those of the Arbequina variety (December) (Table 1). The ripening period occurs earlier in Picual than in Arbequina fruits,^28^ with the Arbequina often not being fully covered by anthocyanins. In their final stage, they achieve purple coloration, in contrast to the black pigmentation of Picual fruits in their final stage.

**Table 1:**
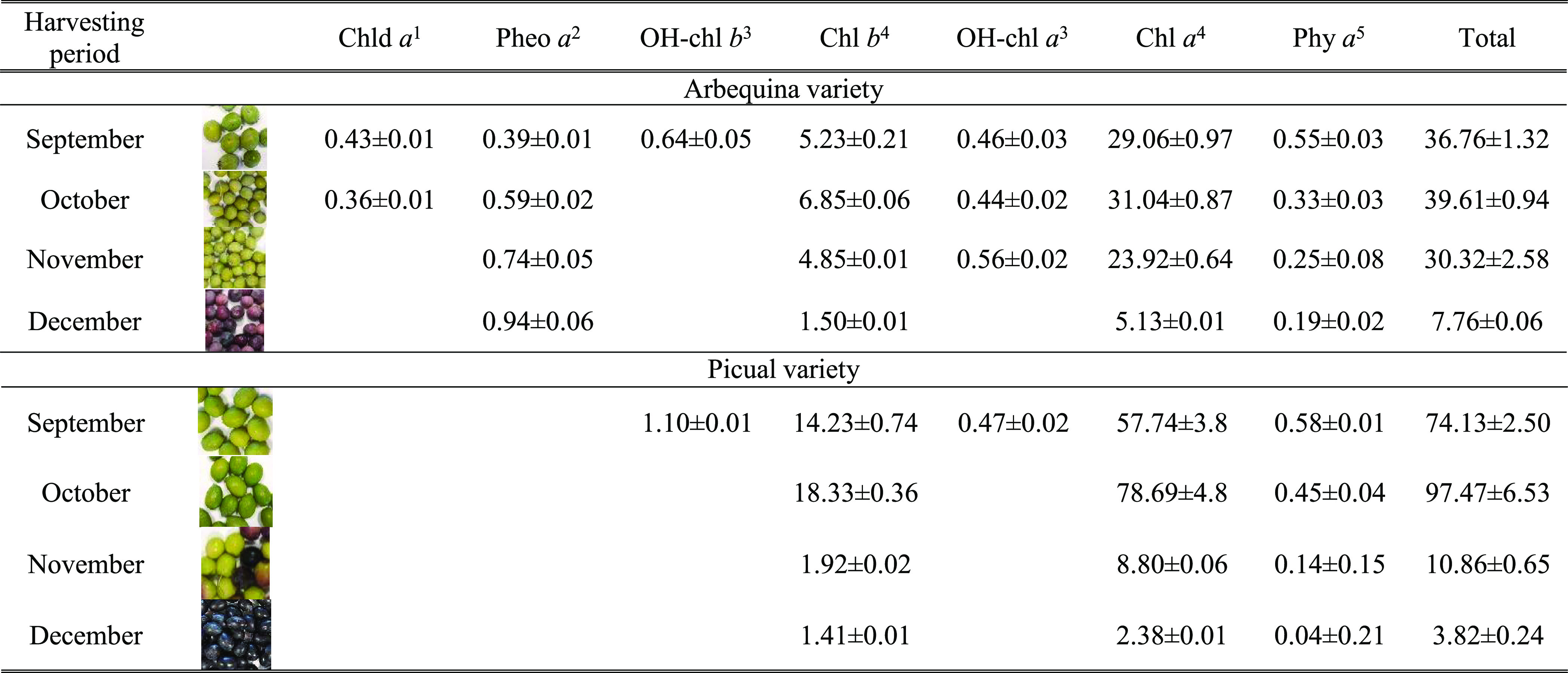
Chlorophyll Composition in Arbequina and Picual Fruits during Different Developmental Stages (mg/kg Dry Weight ± SD)^a^^b^^c^^d^^e^

a Chld *a* stands for chlorophyllide *a.*

b Pheo *a* stands for pheophorbide *a.*

c OH-chl stands for 13^2^-hydroxy-chlorophyll *a*.

d Chl stands for chlorophyll.

e Phy stands for pheophytin.

**Figure 2:**
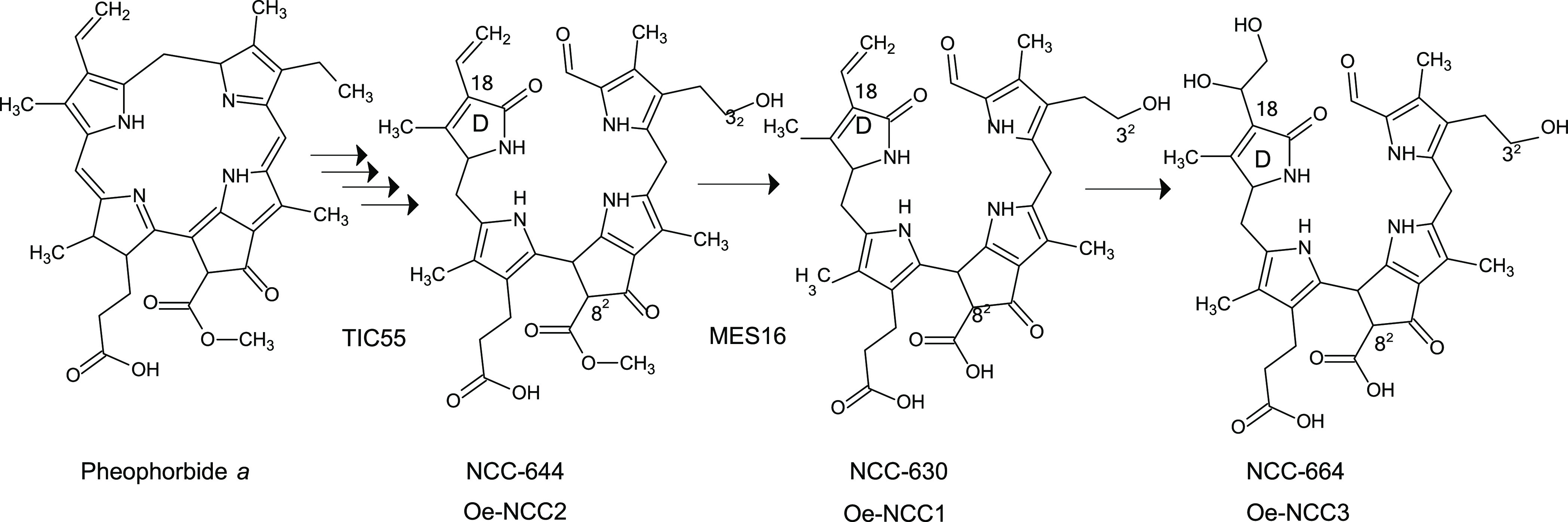
Phyllobilins metabolic pathway in olive fruits.

As shown in Figure 1, pheophorbide *a* was next catabolized to phyllobilins, but given that RCCs and FCCs have fleeting existence,^12^ only the final chlorophyll catabolites accumulated in the vacuole (NCCs and DNCCs) are possible to detect. In olive fruits, two NCCs have been described in Arbequina fruits, namely, Oe-NCC1 and Oe-NCC2.^29^ However, we have renamed them NCC-630 and NCC-644, following the present nomenclature,^12^ describing the molecular weight of each phyllobilin for easier understanding. As shown in Table 2, we have identified the two NCCs already identified in Arbequina fruits and two NCCs in Picual fruits: NCC-644 (in common with Arbequina) and NCC-664. All of them showed the typical UV–vis spectrum with the characteristic maximum at 320 nm (Figure S1).^12^ NCC-630 (or Oe-NCC1) presents an *m/z* of 631.2732 and elemental composition of C_34_H_38_O_4_O_8_. This compound has been identified previously in other senescent leaves, as described in Table 2. Specifically, So-NCC3 (from spinach) was used as a standard to confirm its identification. The retention time, accurate mass, elemental composition, and fragmentation profile were coincident, as was co-elution. NCC-630 presented at C-18 for the vinyl group and at C-3^2^ for hydroxylation, and the methyl ester function at C-8^2^ was hydrolyzed (Figure 2). The same strategy was applied for the identification of the second and common NCC between both varieties, the NCC-644. The *m/z* 645.2936, the elemental composition (C_35_H_40_N_4_O_7_), and the retention time were the same as So-NCC4.^26^ This NCC was used as the standard, which coeluted with NCC-644. The main fragment ions found for NCC-644 were the loss of the D ring and methanol, typical signals for NCCs and specifically for So-NCC4. Consequently, NCC-644 was also present at C-18 for the vinyl group and at C-3^2^ for hydroxylation, and it has a methyl ester function at C-8^2^ (Figure 2). Finally, the new NCC (NCC-664) identified in olive fruits showed an exact mass *m/z* 665.2836 with a predicted elemental composition C_34_H_40_N_4_O_10_, coincident with So-NCC1.^26^ Next, this standard was co-chromatographed, finding that both compounds coeluted at the same retention time. NCC-644 presented at C-18 for the vinyl group, dihydroxylated, at C-3^2^ for hydroxylation, and hydrolyzed the methyl ester function at C-8^2^ (Figure 2). The accumulation of the terminal chlorophyll catabolites in olive fruits coincided with advanced ripening stages (Table 2) when the chlorophyll breakdown is at its maximum, in November and December for Picual, and exclusively in December for Arbequina fruits. The data also showed a higher phyllobilin accumulation in the Picual than in the Arbequina fruits. We also included in the library all the DNCCs already identified in the bibliography,^26^ but the screening of MS data from olives gave no positive results. Therefore, we conclude that ripened olive fruits might not accumulate DNCCs.

**Table 2:** NCCs from Ripe *Olea Europea* L. Fruits (cvs. Arbequina and Picual) Determined by HPLC/ESI-*hr*TOF-MS^*n*^

NCC	Rt (min)	error (ppm)	mSigma	elemental composition	exact mass [M + H]^+^ measured/calculated	MS^2^ fragments	relative amount NCCs		
							October	November	December
Arbequina variety									
NCC-630^a^	47.7	4.7	35.2	C_34_H_38_N_4_O_8_	631.2732/631.2762	613.2636 [M + H-H_2_O]^+^			1067
						508.2040 [M + H-ring D]^+^			
NCC-644^b^	50.3	–2.7	22.9	C_35_H_40_N_4_O_8_	645.2936/645.2919	613.2658 [M + H-CH_3_OH]^+^			24,420
Picual variety									
NCC-644^b^	50.1	–3.1	14.6	C_35_H_40_N_4_O_8_	645.2939/645.2919	522.2176 [M + H-ring D]^+^		15,840	26,848
NCC-664^c^	13.3	–2.8	42.0	C_34_H_40_N_4_O_10_	665.2836/665.2817	509.1707 [M + H-ring D]^+^		15,288	5140

a NCC-630 (Oe-NCC1) = Bn-NCC3 in rape; So-NCC3 in spinach; At-NCC2 in *Arabidopsis thaliana*, Mc-NCC49 in apple; Ej-NCC3 in loquat fruits.^3^

b NCC-644 (Oe-NCC2) = [So-NCC4 in spinach; Cj-NCC1 in *Cercidiphyllum japonicum*; Pc-NCC2 in pears and Md-NCC2 in apples; Sw-NCC58 in peace lily, Ej-NCC4 in loquat fruits;^42^ Vv-NCC-57 in grapevine;^43^ Ob-NCC47 in basil].^44^

c NCC-664 = So-NCC1 in spinach, Mc-NCC26 in apple.^3^

### 3.2 Expression of the Genes Related to Chlorophyll Metabolism

We based the identification of the genes whose expression determines the fate of chlorophyll metabolism in olive fruits on previous work,^30,31^ in which the genome of *O. europaea* was sequenced. To identify the coding sequence (CDS) from the genes *PORA, CLD1, CHL2, SGR1, SGR2, PPH*, and *PAO* (Figure 1) in this species, we created a database that included the corresponding unigenes and coding sequences obtained. For this purpose, the CDS from the homologous genes in *Arabidopsis thaliana* was used as a pattern, and in either case, the unigene or coding sequence corresponding to the CDS of every gene in *O. europaea* was gathered (Table S2).

**Figure 3:**
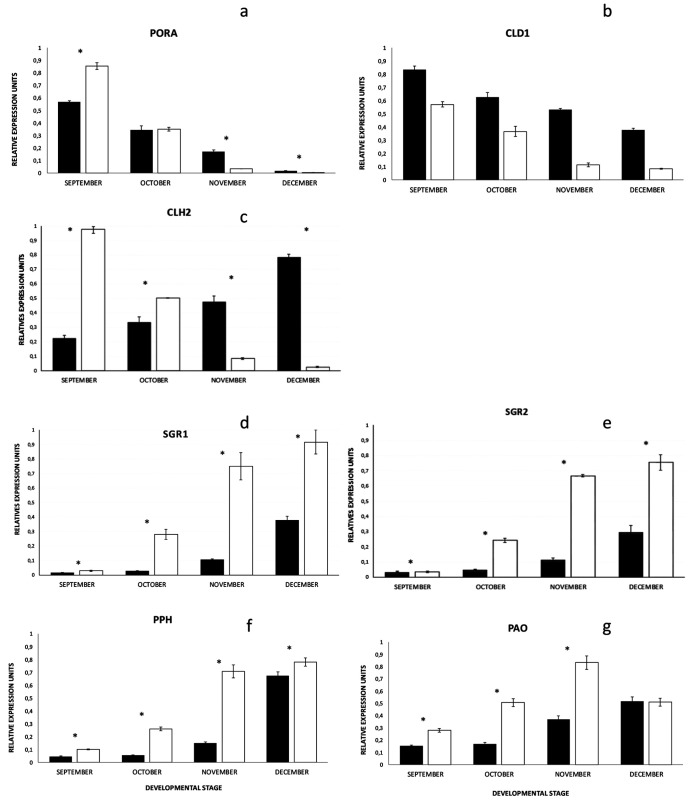
Relative expression pattern of the genes implicated in chlorophyll metabolism of olive fruits of Arbequina (black) and Picual (white) varieties: PORA (a), CLD1 (b), CLH2 (c), SGR1 (d), SGR2 (e), PPH (f), and PAO (g). Statistical differences are marked in asterisks (*).

The expression of *OePORA* (Figures S2–S6) was monitored during the four developmental stages of the olive fruits in both varieties (Figure 3a), with the maximum occurring during the net synthesis of chlorophyll (September) and decreasing throughout their ripening, as expected from a biosynthetic gene. Specifically, the expression of this gene was statistically (*p*-value <0.05) higher in the Picual than in the Arbequina fruits in their early developmental stages (September), concomitant with the maximum chlorophyll synthesis (Table 1). However, the *OeCLD1* (Figures S7–S11) expression (Figure 3b) decreased continuously along with the fruit maturation process (as previously reported for this gene)^32^ and turned out to be higher in the Arbequina than in the Picual fruits (*p*-value <0.05) during the four stages under analysis.

Regarding the historical first chlorophyll degradative enzyme (chlorophyllase), the Q-RT-PCR analysis for *OeCHL2* (Figures S12–S16) displayed an antagonist pattern for both olive fruit varieties (Figure 3c). Although the expression of *OeCHL2* in the Arbequina fruits was parallel with increasing ripening stages, as expected for a catabolic enzyme, in the Picual fruits, the levels of mRNA were inversely proportional to chlorophyll catabolism. However, the expression of *OeSGR1* and *OeSGR2* in olive fruits (Figures S17–S24) shows a typical senescence-gene pattern (Figure 3d,e), increasing throughout the ripening process, as shown by their homologous genes in Arabidopsis.^12^ The expression of both genes was significantly (*p*-value <0.05) higher in the Picual fruits compared with that in the Arbequina fruits, especially toward the end of the ripening process. In parallel, when measuring the expression of *OePPH* (Figures S25–S28) in both varieties, the Picual fruits exhibited consistently higher levels than the Arbequina variety (*p*-value <0.05), although the two displayed a temporal profile concordant with their ripening (Figure 3f). Finally, *AtPAO* expression (Figures S29–S32) was, as with *AtSGR* and *AtPPH*, statistically higher in the Picual than in the Arbequina fruits, except for the last controlled ripening stage, when both showed a similar level of expression (Figure 3g).

### 3.3 Evolution of the Enzymatic Activities Related to Chlorophyll Metabolism

The in vitro determination of chlorophyllase activity during the ripening of olive fruits showed, as expected, a higher dephytylating activity in the Arbequina fruits than in the Picual fruits (Table 3). The activities can reach differences higher than 100 times between varieties, as reported previously,^4,6^ reflecting the exclusive accumulation of dephytylated chlorophyll types (chlorophyllide and pheophorbide) in Arbequina fruits (Table 1). However, although chlorophyllase activity increased concomitantly with the ripening in the Arbequina fruits (Table 1), the dephytylating activity appeared to decrease in the Picual fruits, along with the chlorophyll degradation metabolism.

**Table 3:** Determination of Enzymatic Activities Related to Chlorophyll Metabolism in Arbequina and Picual Fruits during Different Developmental Stages (Medium ± SD)

variety	harvesting period			
	september	october	november	december
chlorophyllase activity (nKat/Kg ap^a^)				
Arbequina	695.40 ± 27.89	798.31 ± 34.83	762.82 ± 65.77	789.47 ± 36.05
Picual	42.10 ± 3.76	11.89 ± 1.21	11.78 ± 1.24	8.01 ± 0.32
PPH activity (nmol/g)				
Arbequina			2.41 ± 0.01	12.49 ± 0.89
Picual	1.68 ± 0.01	7.81 ± 0.53	12.95 ± 0.92	13.52 ± 0.77
PaO activity (fluorescence units)				
Arbequina			144.35 ± 5.20	329.57 ± 10.96
Picual			413 ± 20.38	270 ± 13.84

a ap, acetonic powder.

For the pheophytinase (PPH) dephytylating activity determination, we established the methodology developed initially for Arabidopsis leaves,^9^ adapted to the specific characteristics and metabolism in olive fruits. It has been necessary to increase the reaction time and the enzymatic volumes because olive fruits appear to have lower PPH activity than Arabidopsis leaves. In addition, given that PPH activity is measured by quantifying the amount of pheophorbide *a* formed from pheophytin *a*, it was essential to include different blanks to rest the nonenzymatic formation of pheophorbide. Table 3 shows the net PPH activity measured during the ripening of the two varieties, finding that PPH activity was higher in Picual than in Arbequina fruits and that this activity increases with aging in both varieties. It was impossible to detect PPH activity in the Arbequina fruits during the first developmental stages.

In the chlorophyll degradation pathway, after the dephytylating reaction, the next enzymatic reaction is the opening of the macrocycle, followed by reduction catalyzed by the enzymatic system, PaO-RCCR (Figure 1). The product of the reaction catalyzed by PaO is rapidly channeled to RCCR. Consequently, RCC does not normally accumulate, and the activity determination is the combined measurement of both enzymes.^27^ However, when the PaO activity is very low, and the enzymatic determination is complicated, the combined enzymatic system can be taken advantage of and a control enzyme utilized^23,29^ to allow the activity measurement. The application of this methodology is only possible, thanks to the coordinated enzymatic activity of PaO and RCCR.^27^ The utilization of the same RCCR source with high activity (mature red pepper), in combination with PaO extracts from various developmental stages of olive fruits, allows for the determination of this last enzyme activity in the corresponding fruits (Table 3). Following previous results,^29^ the level of PaO activity in olive fruits only allows its measurement at the end of the ripening stages. Comparing both varieties, PaO activity is higher in Picual fruits than in Arbequina fruits in November (Table 3). Also, while in the Arbequina fruits, the PaO activity increased up to the end of its maturation, in the Picual fruits, the oxygenase activity decreased for the same period. For both varieties, the higher catalytic activity coincided with the periods of considerable chlorophyll degradation. Curiously, the in vivo participating activity of the system PaO-RCCR in olive fruits was shown by the unusual and punctual accumulation of RCC in the Arbequina fruits (Figure S33). Normally, because of the channeling activity of both enzymes, RCC does not accumulate, given that once PaO creates RCC, this compound is rapidly reduced by RCCR to pFCC (Pruzinská et al.^27^). Most likely, in this ripening stage of Arbequina fruits, the combination of a relatively high PaO activity with low RCCR activity has allowed for the momentary accumulation of RCC, which is unusual.

## 4 Discussion

The evolution of chlorophyll content and metabolites during the growth and ripening of olive fruits in different varieties has always been of great interest, given that it determines the color of the corresponding virgin olive oil.^33,34^ As stated previously, olive fruits can be classified as high or low chlorophyll content varieties, and various research projects have dealt with the biochemical implication of chlorophyll’s metabolic enzymes.^4,6^ However, although the biochemical reactions involved in chlorophyll synthesis and breakdown have been deciphered in a broad sense, the chlorophyll metabolism in fruits is mostly unknown because the majority of the research has been developed with senescent leaves. At present, for example, novel dephytylases (PPH, CLD1, and so forth) have been identified, whose exact involvement in chlorophyll metabolism is not yet clear, although it has been proposed that they might play a critical role in the growth of crops.^30^

As explained previously, virgin olive oils, depending on the variety (genetically determined), can be classified as high or low pigmented.^6,21^ To the best of our knowledge, however, the molecular origin of this biosynthetic capacity in olive fruits is unknown. Among all the biosynthetic enzymes, POR A (PORA) has recently been proven to be responsible for the control of chlorophyll production levels,^9^ and it has even been assigned the role of orchestrating the synergy between photosynthetic membrane biogenesis and chlorophyll synthesis.^10^ In olive fruits, the period of net chlorophyll biosynthesis coincides with the maximum levels of *OePORA* expression. Furthermore, the higher chlorophyll biosynthetic capacity of Picual fruits corresponded to a statistically higher level of *OePORA* expression in Picual than in Arbequina fruits (Figure 3). These results, in agreement with other previous results in plants, lead us to propose that PORA is responsible for the higher chlorophyll content in Picual than in Arbequina fruits. The identification of this gene as the determining factor in the chlorophyll biosynthetic capacity in olive fruits could be essential for programs that aim to intensify the color of virgin olive oils.

The highly appreciated Arbequina virgin olive oil exclusively accumulated dephytylated chlorophyll, which has been used as a variety authenticity index. Our data (Table 1) confirm the exceptional accumulation of these metabolites in Arbequina fruits. Until now, the formation of these catabolites (chlorophyllides and pheophorbides) had been associated with the high chlorophyllase activity determined in vitro in the corresponding Arbequina fruits, in comparison with other olive fruit varieties.^4,6^ This assumption was based on the fact that, until recently, the only enzyme believed to be responsible for de-esterification of the phytol chain from the chlorophyll molecule was chlorophyllase. However, CLD1 has been identified in Arabidopsis leaves as an enzyme that can also catalyze this reaction,^19^ but exclusively during the turnover of chlorophylls that continuously work in photosynthetic tissues. Based on these results, for the first time, we have measured the expression of *OeCLD1* in olive fruits (Figure 3b), and as expected, we found higher expression in green tissues.^19^ In addition, the expression in Arbequina fruits was higher than that in Picual fruits during all the controlled stages, concomitant with the exclusive accumulation of dephytylated chlorophylls in those fruits. Furthermore, the quantification of such chlorophyll metabolites had been found during all the developmental stages in olive fruits^6^ (Table 1), more than exclusively in the ripened stages, as would be expected if chlorophyllase were the catalytic enzyme. The metabolite pattern fits better with *OeCLD1* expression than with a senescence enzyme such as chlorophyllase. Moreover, the expression of CHL (Figure 3c) does not agree with the metabolic data (Table 1) of dephytylated chlorophylls. Consequently, based on the results obtained, we propose CLD1 as the enzyme responsible for the accumulation of dephytylated chlorophyll in Arbequina fruits and virgin olive oil, and not chlorophyllase, the enzyme previously thought to be responsible.^6^ The turnover of chlorophyll is an essential physiological mechanism linked to the repair of damaged PSII, whose complete enzymatic mechanism is unknown,^32^ with only CLD1 identified as the first enzyme implied in such a homeostatic process. The participation of CLD1 in chlorophyll turnover has been shown only in Arabidopsis leaves,^19^ and the present results suggest its involvement in the turnover in fruits, in agreement with a previous hypothesis.^35^

The biochemical and molecular analysis of chlorophyll breakdown has been studied mainly in senescent leaves. However, the contradictory results do not allow us to assume that similar reactions will occur during fruit ripening. For example, in broccoli and citrus fruits,^14^ CHL appears to be the enzyme responsible for chlorophyll dephytylation during catabolic breakdown. However, mutants in CHL are still able to degrade chlorophylls.^36^ In olive fruits, we had assumed that CHL was implicated in the first reaction of chlorophyll degradation because chlorophyllase could be enzymatically measured.^4,6^ However, the in vitro measurement of chlorophyllase activity has always been questioned because of the “nonphysiological” conditions required for its determination. Although we have measured chlorophyllase activity during the ripening of Arbequina and Picual fruits (Table 3), the expression pattern of CHL2 (Figure 3c) is not in accordance with the in vitro enzymatic activity measured nor with the in vivo accumulation of dephytylated chlorophyll (Table 1). In fact, CHL2 has been implied in additional functions such as defense,^37^ and recent results showed that CHL2 is not involved in chlorophyll degradation during senescence.^38^ On the contrary, the expression patterns of *OeSGR1/2* and *OePPH* upregulated with ripening are typical senescent genes in accordance with previous results,^35,39^ and are concomitant with progressive chlorophyll degradation (Table 1). Moreover, the higher expression of these genes in Picual than in Arbequina fruits (Figure 3d–f) is in agreement with the highest chlorophyll degradation in Picual fruits (Table 1). This pattern also fits with the profile of PPH activity measured for both varieties (Table 3), in which the activity increased with the ripening stages, and it was higher in Picual than in Arbequina fruits. Consequently, we propose that in olive fruits, the chlorophyll breakdown during the ripening is accomplished by SGR and PPH instead of the classically assumed CHL pathway. Following the chlorophyll degradation pathway (Figure 1), an upregulated *OePaO* (Figure 3g), along with net chlorophyll degradation (Table 1), and parallel PaO activity (Table 3) are indicative of the implication of this enzyme during chlorophyll breakdown in olives. The higher expression of *OePaO* in Picual than in Arbequina fruits, similar to *OeSGR* and *OePPH*, explains the higher chlorophyll degradation in the former.

All the catabolic reactions studied to date correspond to the first part of the PAO/phyllobilin pathway, which encompasses all the reactions involved in the transformation of chlorophyll *b* to chlorophyll *a* and to pFCCs.^12^ The mandatory conversion of chlorophyll *b* to chlorophyll *a* prior to its degradation is supported in olive fruits by the exclusive presence of catabolites from the *a* series (chlorophyllide, pheophorbide, and pheophytin, Table 1). This part of the route occurs inside the chloroplast and is common to all the species investigated thus far. To be accurate, the first reaction modifying the side reaction of phyllobilins (Figures 1 and 2) by a C3^2^-hydrolase (TIC55) should be considered as a part I reaction. From this step onward, the remaining side-chain-modifying reactions over phyllobilins, which take place outside of the chloroplast, should be considered part II reactions. The identification of the three NCCs present in olive fruits based on exact mass, isotopic pattern, MS/MS, UV–vis spectra, product ions obtained during MS^2^ fragmentation, and coelution of authentic standards leads us to propose a phyllobilin biosynthetic pathway in olive fruits.

(Figure 2). NCC-644 is considered the simplest NCC identified so far, given that it only implies the oxidation at C-3^2^ with respect to its substrate pheophorbide *a*. NCC-644 is the common origin for all the NCCs, because the hydroxylation is necessary for the rest of the modifying reactions. This could explain its presence in both varieties. NCC-630 (Figure 2) implies a further modification: the demethylation at C-8^2^. This reaction is catalyzed by the methylesterase 16,^40^ an enzyme belonging to the α/β–hydrolase protein superfamily, whose presence is species-specific. For the first time, terminal chlorophyll catabolites have been analyzed in Picual olive fruits, with the formation of NCC-644 being identified, in common with Arbequina fruits, as well as a new NCC, NCC-664. The new phyllobilin, as shown in Figure 2, originated from NCC-630 but with an additional modification at C18, where the vinyl group was dihydroxylated. Certain experiments indicated that the responsible enzyme could be a dioxygenase;^41^ however, the molecular identification remains elusive. In any case, the massive chlorophyll breakdown in olive fruits toward the end of the ripening process is verified by the quantification of the terminal chlorophyll catabolites (Table 2). In Arbequina fruit, phyllobilins have been identified exclusively at the end of the controlled period (December), whereas in Picual, they occur over several months, corresponding with the earlier ripening period compared with Arbequina fruits.

This multidisciplinary approach has improved our understanding of the origin of the differing chlorophyll content among olive varieties, which ultimately determines the chlorophyll content in the corresponding virgin olive oils. The higher chlorophyll biosynthetic and catabolic capacities among olive varieties are determined at the genetic level, and we have identified what is responsible for these differences. This comprehensive study has also offered a new explanation for the exclusive presence of certain chlorophylls in Arbequina olive oils. In addition to the basic findings, the present results can help in crop improvement programs to obtain the desired olive oils à la carte. It has been shown that fruit ripeness strongly affects oil attributes. In this sense, oils produced by olives at the green stage of maturation (with higher amounts of chlorophylls) showed the highest intensities of positive sensory characteristics (fruity, bitterness, and pungency), major quantities of volatiles from the secondary lipoxygenase (LOX) pathway, and important amounts of natural polar antioxidants and squalene.^45^ Therefore, consumers prefer virgin olive oils with higher chlorophyll content, as well as because virgin olive oil obtained from green olives taste of green fruit, green leaves, or green apples.

## Supporting Information Available

Oligonucleotide primer sets used for Q-RT-PCR analysis (Table S1). The unigene or coding sequence, corresponding to the CDS of every gene in *Olea europaea* (Table S2). UV–vis of NCC-644 (Figure S1). Full-length coding sequence of *OePORA* (Figure S2), *OeCLD1* (Figure S7), *OeCHL2* (Figure S13)*, OeSGR1* (Figure S17), *OeSGR2* (Figure S18), *OePPH* (Figure S25), and *OePAO* (Figure S29). Full-length protein sequence of OePORA (Figure S3), OeCLD1 (Figure S8), OeCHL2 (Figure S14), OeSGR1 (Figure S19), OeSGR2 (Figure S20), OePPH (Figure S26) and OePAO (Figure S30). Full-length coding sequence alignment of *AtPORA* and *OePORA* (Figure S4), *AtCLD1* and *OeCLD1*(Figure S9) and *AtCHL2* and *OeCHL2* (Figure S12). Amino acid alignments of the predicted proteins of OePORA with AtPORA (Figure S5), OeCLD1 with AtCLD1 (Figure S10), OeCHL2 with AtCHL2 (Figure S15), OeSGR1 with AtSGR1 (Figure S21), OeSGR2 with AtSGR2 (Figure S22), OePPH with AtPPH (Figure S27), and OePAO with AtPAO (Figure S31). Domains in the predicted proteins of OePORA (Figure S6), OeCLD1 (Figure S11), OeCHL2 (Figure S16), OeSGR1 (Figure S23), OeSGR2 (Figure S24), OePPH (Figure S28), and OePAO (Figure S32). UV–vis spectra of an RCC formed during the in vitro PaO activity measurement in Arbequina fruits (Figure S33) (PDF)

## Abbreviations Used

CHL: chlorophyllase
CLD1: chlorophyll dephytilase1
DNCCs: dioxo-NCCs
FCC: fluorescent chlorophyll catabolite
NCC: nonfluorescent chlorophyll catabolite
PaO: pheophorbide *a* oxygenase
POR(A): protochlorophyllide oxidoreductase A
PPH: pheophytinase
RCC: red chlorophyll catabolite
RCCR: red chlorophyll catabolite reductase
SGR: stay-green.
