# Supplementary material for: Multiomics Approach To Decipher the Origin of Chlorophyll Content in Virgin Olive Oil
Source: J Agric Food Chem. 2022 Mar 15;70(12):3807–17. doi: 10.1021/acs.jafc.2c00031 (PMC8972264; doi:10.1021/acs.jafc.2c00031)

Appendix

Table S1. Primers sequences.

Each primer pairs generated a single amplicon of 100-200 bp; primers had compatible melting temperatures (within 5 °C). Only primer pairs that produced the expected amplicon and showed similar efficiency were selected for use.

**Name**	**Sequence**
18SL	5' - AAACGGCTACCACATCCAAG - 3'
18SR	5' - CCTCCAATGGATCCTCGTTA - 3'
GAPDHL	5' - ACAGCTCCTGGTAAGGGTGA - 3'
GAPDHR	5' - GGCTTGCGTCAAGAAGTCTC - 3'
UBQ2L	5' - AATGAAGTCTGTCTCTCCTTTGG - 3'
UBQ2R	5' - AAGGGAAATCCCATCAACG - 3'
PORAL	5' - CCCTGTACCCTGGTTGCATT - 3'
PORAR	5' - CCTGTTTCCGGCTTCTTCCT - 3'
PAOL	5' - CACTCCTACTGCCAGCACTT - 3'
PAOR	5' - CCAGGTCCCGATTCAGTAGC - 3'
PPHL	5' - TTCCCCACCAGTGCTTTTCC - 3'
PPHR	5' - TACGCCGCAAAGTTGGATCT - 3'
CLD1L	5' - TGCCTTCTCGACTGCCAAAT - 3'
CLD1R	5' - TAAGATGCGGGGAGATTGGC - 3'
CLH2L	5' - GCACCCCAGTTATACAGCGT - 3'
CLH2R	5' - CCGGTCGAACATTAGCTGGA - 3'
SGR1L	5' - ATGAAGTGGTGGCAGAGTGG - 3'
SGR1R	5' - TGAGCCTAGCACAGAGATCCA - 3'
SGR2L	5' - CCTCTAAGCTCACTCTTGCCA - 3'
SGR2R	5' - TTCCACTCTGCCACCACTTC - 3'

Table S2: Unigen or coding sequence for the CDS in *O. europaea*.

**Gene**	**Unigen sequence**	**Coding sequence**
PORA	--	OE6A003327P2
CLD1	--	OE6A075433P4
CLH2	--	OE6A061932P1
PPH	Unigene039760	--
PAO	--	OE6A054840P1
SGR1	Unigene040952	--
SGR2	Unigene042170	--

Figure S1. UV-Vis of NCC-644.

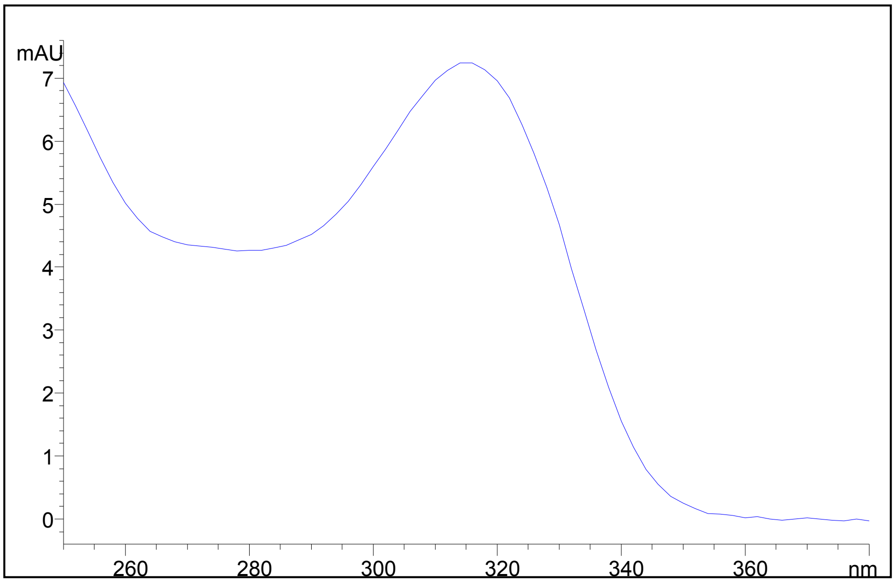

Figures S2-S5: The OePORA full-length coding sequence (Fig. S2) with 1182 bp encoded a 393 amino acid protein (Fig. S3) with a calculated mass of 43.75 KDa. Both deduced sequences shared an identity higher than 90% with the AtPORA (Fig. S4) and a similarity of 88% with AtPORA (Figure S5) respectively. OePORA includes the Rossman-fold domain characteristic of the short-chain dehydrogenases/reductases family, of which PORA is known to be a member (Fig. S6). The amino acid sequence also includes the common structural features of this family, a glycine-rich GXXXGXG NADPH-binding motif (Garrone et al., 2015).

Figure S2: *OePORA* full-length coding sequence.

ATGGCTCTACAGGCTGCTGCCATTCTCCCCTCTGCATTGGCCATTCACAAAGAGG
GAAAAAATAGTGCATCCTTCAAGGACTCTAACCTCTTTGGAATTTCAATCTCAGAC
CATGTCAAGGCTGACAAGAGAGAATTCATTTCGAGGAAGTTCTCCATCGGGGCTA
TCAGAGCTGAGGCAGTGGCTACCTATCCAGTTTTCACCCCTGCAACAGTTCAAGG
GAAGAAAACACTAAGAAAGGGCACTGTGGTGATCACTGGAGCCTCCTCGGGATTA
GGCCTGGCGACGGCAAAGGCTCTGGCTGAAACCGGAAAATGGCACGTAATTATG
GCGTGCAGGGACTTCCTCAAGGCTGAGAGGGCAGCAAAGTCAGTTGGAATGTCT
AAGGAAAATTACACCATTATGCATTTAGACCTTGCTTCCTTTGACAGTGCGAGGCA
GTTTGTCGAAAACTTCAAGCAATCTGGCCGACCCCTCGACGTCTTGGTCTGCAAT
GCTGCTGTCTATCAGCCAACTGCTAGGGAACCTTCATTCACAGCTGATGGGTTTG
AGCTGAGTGTTGGGACTAACCATCTTGGTCATTTCCTTCTTTCAAGATTGTTGCTC
GATGACATGAAGCAGTCTGATTATCCATCGAAGAGACTCATAATTGTCGGCTCAAT
TACAGGGAATACAAATACACTGGCTGGAAATGTACCTCCAAAGGCGAATCTTGGG
GATTTGAGGGGCCTCCAAGGGGGATTGAATGGATTAAACACCTCGTCCATGATTG
ACGGTGGAGATTTCGATGGAGCAAAGGCGTACAAGGATAGCAAGGTGTGCAATAT
GCTAACGATGCAAGAGTTTCACCGTCGTTACCATGAAGAAACTGGGATCACATTT
GCTTCCCTGTACCCTGGTTGCATTGCCACAACAGGCCTGTTTAGGGAGCACATCC
CCTTGTTTAGGCTCCTTTTCCCTCCATTTCAAAAGTTCATCACCAAGGGGTTCGTC
TCAGAGGAAGAAGCCGGAAACAGGCTTGCACAGGTTGTAAGTGATCCAAGTTTAA
CAAAGTCAGGAGTTTACTGGAGTTGGAACAAGGATTCGGCTTCATTCGAGAACCA
GTTGTCTCAAGAAGCCAGTAATGCCGATAAAGCTCGTAAAGTATGGGAAATCAGT
GAGAAACTAGTTGGCTTGGCTTGA

Figure S3: OePORA full-length protein sequence.

M A L Q A A A I L P S A L A I H K E G K N S A S F K D S N L F G I S I S D H V K A D
K R E F I S R K F S I G A I R A E A V A T Y P V F T P A T V Q G K K T L R K G T V V
I T G A S S G L G L A T A K A L A E T G K W H V I M A C R D F L K A E R A A K S V
G M S K E N Y T I M H L D L A S F D S A R Q F V E N F K Q S G R P L D V L V C N
A A V Y Q P T A R E P S F T A D G F E L S V G T N H L G H F L L S R L L L D D M K
Q S D Y P S K R L I I V G S I T G N T N T L A G N V P P K A N L G D L R G L Q G G
L N G L N T S S M I D G G D F D G A K A Y K D S K V C N M L T M Q E F H R R Y H
E E T G I T F A S L Y P G C I A T T G L F R E H I P L F R L L F P P F Q K F I T K G F
V S E E E A G N R L A Q V V S D P S L T K S G V Y W S W N K D S A S F E N Q L S
Q E A S N A D K A R K V W E I S E K L V G L A

Figure S4: *AtPORA* and *OePORA* full-length coding sequence alignment. Conserved nucleotides are marked with an asterisk. Pink shades indicate conserved regions. Yellow and green shades and dashed lines indicate non-conserved or missing regions.

```
Arabidopsis    ATGGCCCTTCAAGCTGCTTCTTTGGTCTCCTCTGCTTTCTCTGTCCGCAAAGATGGAAAATTAAATGCT
Olea           ATGGCTCTACAGGCTGCTGCCATTCTCCCCTCTGCATTGGCCATTCACAAAGAGGGAAAAAATA-----

cons           ***** ** ** ****** *  *  ** ******* **  *  * * ****** ******   *     

Arabidopsis    TCAGCATCATCATCATTCAAAGAGTCTAGTCTGTTCGGTGTTTCACTTTCGGAGCAAAGCAAAGCTGAC
Olea           ---GTG-C---ATCCTTCAAGGACTCTAACCTCTTTGGAATTTCAATCTCAGACCATGTCAAGGCTGAC

cons              *   *   *** ***** ** ****  ** ** **  ***** * ** ** **   *** ******

Arabidopsis    TTTGTCTCTTCCTCATTGAGATGCAAGAGGGAACAGAGCTTGAGGAAT---AATAAAGCGATTATTCGA
Olea           AAGAGAGAAT--TCATT----------------------TCGAGGAAGTTCTCCATCGGGGCTATCAGA

cons                    *  *****                      * ******       *  * *  ***  **

Arabidopsis    GCTCAAGCAATCGCGACTTCAACTCCATCAGTCACAAAATCTTCCTTAGACCGCAAGAAAACACTTAGA
Olea           GCTGAGGCAGTGGC---TACCTATCCAGTTTTCACCCCTGCAACAGTTCAAGGGAAGAAAACACTAAGA

cons           *** * *** * **   * *   ****    ****     *  *  *  *  * *********** ***

Arabidopsis    AAAGGAAACGTGGTTGTCACGGGAGCTTCTTCAGGGCTAGGTTTAGCAACGGCAAAGGCATTAGCCGAG
Olea           AAGGGCACTGTGGTGATCACTGGAGCCTCCTCGGGATTAGGCCTGGCGACGGCAAAGGCTCTGGCTGAA

cons           ** ** *  *****  **** ***** ** ** **  ****  * ** ***********  * ** ** 

Arabidopsis    ACAGGTAAATGGCACGTGATAATGGCGTGCAGAGACTTCCTCAAGGCTGAGAGAGCCGCTCAATCTGCA
Olea           ACCGGAAAATGGCACGTAATTATGGCGTGCAGGGACTTCCTCAAGGCTGAGAGGGCAGCAAAGTCAGTT

cons           ** ** *********** ** *********** ******************** ** **  * ** *  

Arabidopsis    GGGATGCCTAAGGACAGCTACACTGTGATGCATTTGGACTTGGCGTCTTTGGACAGCGTGAGGCAGTTT
Olea           GGAATGTCTAAGGAAAATTACACCATTATGCATTTAGACCTTGCTTCCTTTGACAGTGCGAGGCAGTTT

cons           ** *** ******* *  *****  * ******** *** * ** ** ** ***** * **********

Arabidopsis    GTTGATAACTTCAGGCGAGCTGAGATGCCTCTCGATGTGTTGGTCTGCAATGCCGCAGTCTATCAGCCA
Olea           GTCGAAAACTTCAAGCAATCTGGCCGACCCCTCGACGTCTTGGTCTGCAATGCTGCTGTCTATCAGCCA

cons           ** ** ******* ** * ***     ** ***** ** ************** ** ************

Arabidopsis    ACGGCTAATCAACCTACTTTCACTGCTGAAGGGTTTGAGCTTAGCGTTGGGATAAACCATTTGGGCCAC
Olea           ACTGCTAGGGAACCTTCATTCACAGCTGATGGGTTTGAGCTGAGTGTTGGGACTAACCATCTTGGTCAT

cons           ** ****   ***** * ***** ***** *********** ** *******  ****** * ** ** 
```

```
Arabidopsis    TTTCTTCTTTCAAGATTGTTGATTGATGACTTGAAGAACTCCGATTATCCATCAAAACGTCTCATCATT
Olea           TTCCTTCTTTCAAGATTGTTGCTCGATGACATGAAGCAGTCTGATTATCCATCGAAGAGACTCATAATT

cons           ** ****************** * ****** ***** * ** *********** **  * ***** ***


Arabidopsis    GTTGGATCCATAACCGGAAACACTAATACATTGGCTGGTAATGTACCTCCAAAGGCTAATCTTGGTGAT
Olea           GTCGGCTCAATTACAGGGAATACAAATACACTGGCTGGAAATGTACCTCCAAAGGCGAATCTTGGGGAT

cons           ** ** ** ** ** ** ** ** ****** ******* ***************** ******** ***


Arabidopsis    TTGAGGGGACTAGCAGGCGGGTTGAACGGGCTAAACAGCTCGGCGATGATAGATGGAGGAGATTTTGTT
Olea           TTGAGGGGCCTCCAAGGGGGGATTGAATGGATTAAACACCTCGTCCATGATTGACGGTGGAGATTTCGAT

cons           ******** **    *** ** ***** **  ****** **** * ***** ** ** ******** * *


Arabidopsis    GGCGCAAAGGCGTATAAAGATAGCAAAGTCTGCAACATGTTGACAATGCAGGAGTTTCATAGGCGTTTC
Olea           GGAGCAAAGGCGTACAAGGATAGCAAGGTGTGCAATATGCTAACGATGCAAGAGTTTCACCGTCGTTAC

cons           ** *********** ** ******** ** ***** *** * ** ***** ********  * **** *


Arabidopsis    CATGAAGACACCGGCATAACTTTTGCTTCCCTTTACCCCGGTTGTATTGCAACGACTGGTTTGTTCAGA
Olea           CATGAAGAAACTGGGATCACATTTGCTTCCCTGTACCCTGGTTGCATTGCCACAACAGGCCTGTTTAGG

cons           ******** ** ** ** ** *********** ***** ***** ***** ** ** **  **** **


Arabidopsis    GAGCATATTCCTCTTTTCCGTACCCTCTTCCCTCCTTTCCAGAAGTACATCACAAAAGGTTACGTCTCC
Olea           GAGCACATCCCCTTGTTTAGGCTCCTTTTCCCTCCATTTCAAAAGTTCATCACCAAGGGGTTCGTCTCA

cons           ***** ** **  * **  *   *** ******** ** ** **** ****** ** ** * ******


Arabidopsis    GAGTCAGAGGCTGGGAAAAGACTTGCTCAGGTGGTGGCTGATCCAAGCTTGACGAAGTCGGGAGTGTAT
Olea           GAGGAAGAAGCCGGAAACAGGCTTGCACAGGTTGTAAGTGATCCAAGTTTAACAAAGTCAGGAGTTTAC

cons           ***  *** ** ** ** ** ***** ***** **   ********* ** ** ***** ***** **


Arabidopsis    TGGAGCTGGAACAAGACCTCGGCTTCATTTGAGAATCAGCTGTCTCAAGAAGCTAGCGATGTCGAGAAG
Olea           TGGAGTTGGAACAAGGATTCGGCTTCATTCGAGAACCAGTTGTCTCAAGAAGCCAGTAATGCCGATAAA

cons           ***** *********   *********** ***** *** ************* **  *** *** **


Arabidopsis    GCTCGTAGAGTTTGGGAAGTCAGCGAGAAGCTCGTAGGCTTGGCCTAA
Olea           GCTCGTAAAGTATGGGAAATCAGTGAGAAACTAGTTGGCTTGGCTTGA

cons           ******* *** ****** **** ***** ** ** ******** * *
```

Figure S5: Amino acid alignment of predicted protein OePORA with AtPORA. Conserved residues are marked with an asterisk. Dots indicate residues with similar properties. Pink shades indicate conserved regions. Yellow and green shades and dashed lines indicate non-conserved or missing regions. The common structural features of the short-chain dehydrogenases/reductases family, a glycine-rich GXXXGXG NADPH-binding motif is underlined with a red box (Garrone et al., 2005).

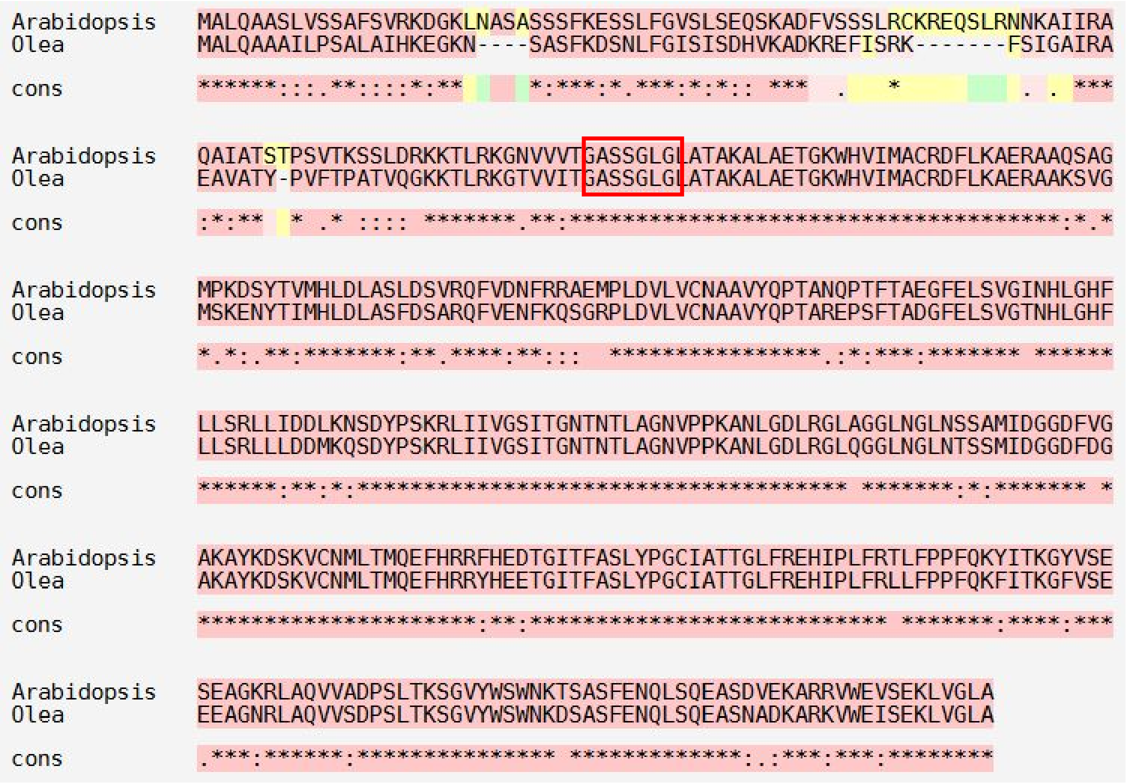

Figure S6: Domains in the predicted protein OePORA

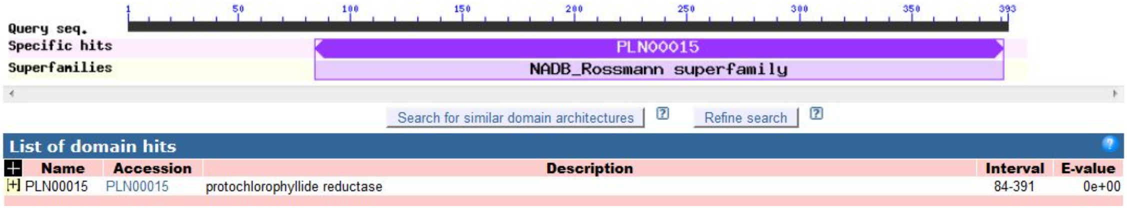

Figures S7-S11: The OeCLD1 full-length coding sequence (Fig. S7) consisted of 1128 bp, which encoded a 375 amino acid protein (Fig. S8) with a calculated mass of 41 KDa. Both deduced sequences shared an identity higher than 90% with the AtCLD1 (Fig. S9) and a similarity higher than 72% with AtCLD1(Figure S10) respectively. The amino acid sequence of OeCLD1 includes the common domain of the α/β-hydrolases superfamily protein where CLD1 belongs (Fig. S11). OeCLD1 also contains the short peptide sequence proposed as the active site for CLD1: G-N-S-V-G (Lin et al. 2016).

Figure S7: *OeCLD1* full-length coding sequence.

ATGGGCACGGCAGTAAATCTTGCTGCAACCTATATTTTCCTTCCAACGCATTCTCC
TTCAATTACTCTACCAAAACAGTACAATCCCATCTGCAGTTCACAAAAATTTTCCAA
AATTTCTTCCTACAGTAAATTCAAGAATCCCATTAGTGTAATTAATGCCTCTACTGC
TTCTGCGTCAACTGAAAAAAGGGAGGTGTTGTTGCCATTGGAAGTGAGTGAGATA
AATGAAAAGTGCAAAAAATGGGTGTGGAAAGGTTACACCATTAACTATTTTGTTTA
CCCAGGAGGCAATAATGATTCTTCAAATCCGCCTCTTCTGCTAATTCACGGTTTTG
GTGCCTCCATTGCTCATTGGCGCAGGAATATTCCGACTCTGGCTCAAAGTTATACA
GTTTACGCCATTGACCTCCTTGGTTTTGGTGCTTCAGATAAGCCAGCGGGCTTTG
CATATTCCATGGAAGTATGGGCTGAGATGATATTGGACTTCTTAGACGAAATTGTT
CAAAGGCCGGCGATACTAATGGGGAACTCTGTTGGAAGTCTTGCTTGCGTAATTG
CCGCTGCAGAGTCTACTAGGACCCTAGTTCGAGGGCTCGTTCTGTTAAATTGTGC
TGGCGGCATGAACAACAAGGCGATTGTTGATGACTGGAGGATAAAGCTACTGTTG
CCTTTGCTTTGGTTTGTCGACTTCTTATTGAAGCAAAAAGGGATTGCATCGTATTT
GTTTGATCGTGTTAGACAAAGAGACAGTCTGAGGAACATCTTGTCATCCGTCTATG
GCAATAAGGATAATGTCGATGAGGGTCTTGTGGATATTATTAAGAAACCAGCAGAA
GATGAAGGGGCTCTTGATGCTTTTGTTTCCATTGTAACTGGCCCCCCTGGGCCGA
ATCCAATGCAATTAATTCCGAAAATCAACTTACCTGTCCTCGTGTTATGGGGTGAT
CAAGATCCATTTACACCGATTGATGGACCTGTTGGTAAATACTTCTCGTCGTTGCC
TTCTCGACTGCCAAATGTGAACCTCTTTTTGTTGGAAGGCGTTGGTCATTGCCCTC
ATGATGACAGGCCTGACTTAGTGCACGAGAATTTGCTTCCTTGGCTAGCCAATCT
CCCCGCATCTTAA

Figure S8. OeCLD1 full-length protein sequence.

MGTAVNLAATYIFLPTHSPSITLPKQYNPICSSQKFSKISSYSKFKNPISVINASTASAST
EKREVLLPLEVSEINEKCKKWVWKGYTINYFVYPGGNNDSSNPPLLLIHGFGASIAHW
RRNIPTLAQSYTVYAIDLLGFGASDKPAGFAYSMEVWAEMILDFLDEIVQRPAILMGNS
VGSLACVIAAAESTRTLVRGLVLLNCAGGMNNKAIVDDWRIKLLLPLLWFVDFLLKQKG
IASYLFDRVRQRDSLRNILSSVYGNKDNVDEGLVDIIKKPAEDEGALDAFVSIVTGPPG
PNPMQLIPKINLPVLVLWGDQDPFTPIDGPVGKYFSSLPSRLPNVNLFLLEGVGHCPH
DDRPDLVHENLLPWLANLPAS

Figure S9. *AtCLD1* and *OeCLD1* full-length coding sequence alignment. Conserved nucleotides are marked with an asterisk. Pink shades indicate conserved regions. Yellow and green shades and dashed lines indicate non-conserved or missing regions.

```
Arabidopsis    ATGAGA---GCTCTAACATGG------ACGGCAATGTCGCCG---------------CCGGTGATGTCA
Olea           ATGGGCACGGCAGTAAATCTTGCTGCAACCTATATTTTCCTTCCAACGCATTCTCCTTCAATTACTCTA

cons           *** *    **  ***           **    ** *  *                  *  * *    *

Arabidopsis    C-------GGACGGCGACTTCT-------ACGGTTAACTTACGACGTATCAGCCTACGGAGAGATCGCG
Olea           CCAAAACAGTACAATCCCATCTGCAGTTCACAAAAATTTTCCAAAATTTCTTCCTACAGTAAATTCAAG

cons           *       * **     * ***       **    *  ** * *  * **  ***** *  *  **  *

Arabidopsis    TCT-GTGTTAGAGCCACGGCTTCGTCTAGCGCCACGGTTTCCGGCGGAGGAGT------AGTAGAGGCG
Olea           AATCCCATTAGTGTAATTAATGCCTCTACTGCTTCTGCGTCAACTGAAAAAAGGGAGGTGTTGTTGCCA

cons             *    **** *  *    * * ****  **  * *  **    * *  *          *   * *

Arabidopsis    GTAGAATTGGCAGAGATAGGAGAAAGAAGCAAGAAATGGAAGTGGAAAGGAGAATATTCTGTTAACTAC
Olea           TTGGAAGTGAGTGAGATAAATGAAAAGTGCAAAAAATGGGTGTGGAAAGGT---TACACCATTAACTAT

cons            * *** **   ******   ****   **** ******  *********    **  *  *******

Arabidopsis    TTTGTCAAAGATTCGCCGGAGGAAGTTACTCCGGCGAGTCAAACTGTTCTTTTGGTTCATGGCTTTGGT
Olea           TTTGTTTA---CCCAGGAGGCAATAATGATTCTTCAAATCCGCCTCTTCTGCTAATTCACGGTTTTGGT

cons           *****  *     *    *   *   *  * *  * * **   ** ****  *  **** ** ******

Arabidopsis    GCTTCTATTCCTCACTGGCGAAGGAACATAAATGCTTTGTCTAAAAACCATACAGTGTATGCAATTGAT
Olea           GCCTCCATTGCTCATTGGCGCAGGAATATTCCGACTCTGGCTCAAAGTTATACAGTTTACGCCATTGAC

cons           ** ** *** **** ***** ***** **     ** ** ** ***   ******* ** ** *****

Arabidopsis    CTTCTCGGGTTTGGTGCTTCGGATAAGCCACCTGGTTTTAGCTATACCATGGAGTCATGGGCTGAGTTG
Olea           CTCCTTGGTTTTGGTGCTTCAGATAAGCCAGCGGGCTTTGCATATTCCATGGAAGTATGGGCTGAGATG

cons           ** ** ** *********** ********* * ** ***   *** *******   ********** **

Arabidopsis    ATACTCAACTTCTTGGAGGAAGTGGTTCAGAAACCGACTATTTTGATTGGAAACTCTGTTGGAAGCCTT
Olea           ATATTGGACTTCTTAGACGAAATTGTTCAAAGGCCGGCGATACTAATGGGGAACTCTGTTGGAAGTCTT

cons           *** *  ******* ** *** * ***** *  *** * **  * ** ** ************** ***

Arabidopsis    GCTTGTGTCATCGCTGCCTCAGGTACTAAGTTTCTCATATATTTGGAAAAAAAAACAGAATCACGAGGA
Olea           GCTTGCGTAATTGC---CGCTG---CAGAGT--CT-----ACTAGGA----------------------

cons           ***** ** ** **   * * *   *  ***  **     * * ***
```

```
Arabidopsis    GATCTGGTCAAAGGTCTTGTTCTATTGAATTGTGCTGGTGGTATGAACAACAAAGCTGTCTTTGATGAC
Olea           -CCCTAGTTCGAGGGCTCGTTCTGTTAAATTGTGCTGGCGGCATGAACAACAAGGCGATTGTTGATGAC

cons              ** **   *** ** ***** ** *********** ** *********** **  *  ********

Arabidopsis    TGGAGAATCAAGCTACTGATGCCTTTACTCTTACTTATCGACTTCTTACTCAAGCAAAGAGGAATTGCT
Olea           TGGAGGATAAAGCTACTGTTGCCTTTGCTTTGGTTTGTCGACTTCTTATTGAAGCAAAAAGGGATTGCA

cons           ***** ** ********* ******* ** *   ** *********** * ******* *** *****

Arabidopsis    TCTGCACTCTTCAACCGTGTTAAAGACAGGGAAAATCTGAAGAACATCTTGACAAATGTTTACGGGAAC
Olea           TCGTATTTGTTTGATCGTGTTAGACAAAGAGACAGTCTGAGGAACATCTTGTCATCCGTCTATGGCAAT

cons           **     * **  * ******* * * ** ** * ***** ********** **   ** ** ** **

Arabidopsis    AAGGACAATGTAGATGACACCCTTGTAGAGATCATTGCTGGACCAGCAAATACCGAAGGTGCACTAGAT
Olea           AAGGATAATGTCGATGAGGGTCTTGTGGATATTATTAAGAAACCAGCAGAAGATGAAGGGGCTCTTGAT

cons           ***** ***** *****    ***** ** ** ***     ******* *    ***** ** ** ***

Arabidopsis    GCTTTTGTTTCAATCTTAACGGGTCCTCCTGGACCAAATCCGATTAAGCTGATACCGGAAATAACCAAA
Olea           GCTTTTGTTTCCATTGTAACTGGCCCCCCTGGGCCGAATCCAATGCAATTAATTCCGAAAATCAACTTA

cons           *********** **  **** ** ** ***** ** ***** **  *  * ** *** **** * *  *

Arabidopsis    CCGGTTCTTGTCTTATGGGGAGATCAAGATGGACTAACCCCTCTTGATGGTCCAGTAGGTAAGTACTTC
Olea           CCTGTCCTCGTGTTATGGGGTGATCAAGATCCATTTACACCGATTGATGGACCTGTTGGTAAATACTTC

cons           ** ** ** ** ******** *********  * * ** **  ******* ** ** ***** ******

Arabidopsis    ACTTCCCTTCCGGATCAGTTACCTAACTTCAACCTCTATGTTCTACAAGGCGTTGGACATTGCCCGCAA
Olea           TCGTCGTTGCCTTCTCGACTGCCAAATGTGAACCTCTTTTTGTTGGAAGGCGTTGGTCATTGCCCTCAT

cons            * **  * **   **   * ** **  * ******* * *  *  ********** ******** **

Arabidopsis    GATGATCGTCCGGATCTTGTCCATGAGCGTCTCCTTCCATGGCTGGCTCAACTTTCTTCCACCTAG
Olea           GATGACAGGCCTGACTTAGTGCACGAGAATTTGCTTCCTTGGCTAGCCAATCTCCCCGCATCTTAA

cons           *****  * ** **  * ** ** ***  * * ***** ***** **  * **  *  *  * **
```

Figure S10. Amino acid alignment of predicted protein OeCLD1 with AtCLD1. Conserved residues are marked with an asterisk. Dots indicate residues with similar properties. Pink shades indicate conserved regions. Yellow and green shades and dashed lines indicate non-conserved or missing regions. The active site G-N-S-V-G (Lin et al. 2016) is underlined with a red box.

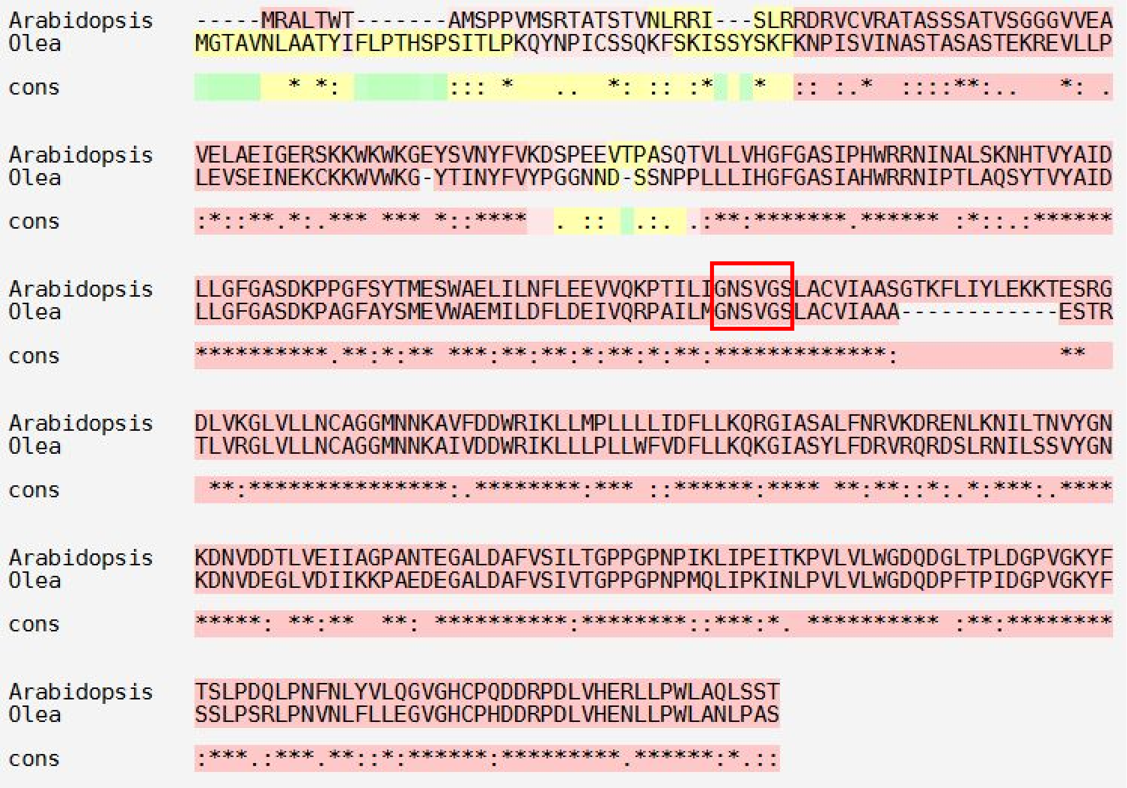

Figure S11: Common domain of the α/β-hydrolases superfamily protein OeCLD1

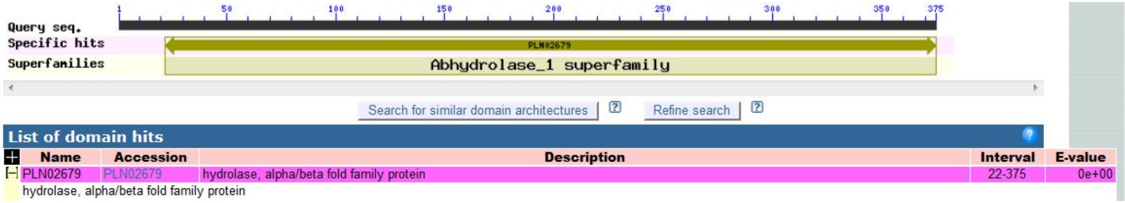

Figures S12-S16: It has been described the existence of two copies of AtCHL (Tsuchiya et al., 1999). Group I of CHLs has been related to a response to environmental stimuli as plant defense (Lira et al., 2014), while only group II of CHLs contains a transit peptide to the chloroplast (Jacob-Wilkson et al., 1999). We looked at the genome of *Olea europaea* for both genes, finding no positive match for OeCHL1, only for OeCHL2 (Fig. S12). The existence of only one copy of CHL in the genome is common among different species (Jacob-Wilkson et al., 1999; Tsuchiya et al., 1999). OeCHL2 full-length coding sequence with 957 bp (Fig. S13) encoded a 318 amino acid protein (Fig S.14) with a calculated mass of 34.84 KDa. OeCHL2 shares almost 80% similarity with the AtCHL2 and belongs to the α/β hydrolase superfamily following the domain study (Fig. S15), as expected for this serine lipase. OeCHL2 also exhibits the conserved catalytic motif (GHSRRG, Figure S16) among the serine hydrolases, and specifically in the identified chlorophyllases (Tsuchiya et al. 2003).

Figure S12: *AtCHL2* and *OeCHL2* full-length coding sequence alignment. Conserved nucleotides are marked with an asterisk. Pink shades indicate conserved regions. Yellow and green shades and dashed lines indicate non-conserved or missing regions.

```
Arabidopsis   ATGTCCTCTTCTTCATCAAGAAACGCCTTTGAAGATGGCAAATACAAATCAAATCTCTTAACCTTGGA-
Olea          ATGAATTCTTCATCTACAACTCCCACCTTATCTTCTTCTTCCTC-TAA-AAATGCTTTTGATATTGGAA

cons          ***   ***** **  ***    * ****      *      *   **  **  ** ** *  *****

Arabidopsis   CTCATC--ATCTCGTTGCTG---------CAAAATAACACCGTCTTCTAGAGCTTCA-CCGTC-TCCGC
Olea          ATCATTTAATTAAGCTGATTAAAATTGAGCCTAAAATAAGCAATAACAGTGGTTCAGTCTGTTTTCCTC

cons           ****   **   * ** *          *  ** *  * *     *    * *    * **  *** *

Arabidopsis   CAAAGCAGCTGTTGGTGGCTACGCCGGTGGAGGAAGGAGATTATCCGGTGGTGATGCTCCTCCATGGTT
Olea          CGAAGCCACTTTTAATTGGGACACCAACAGAATCTGGAGTTTTCCCAGTAGTTATACTCCTTCATGGTT

cons          * ****  ** **  * *  ** **    **    **** **  ** ** ** ** ***** *******

Arabidopsis   ACCTTCTCTACAACTCCTTCTATTCTCAGCTTATGTTGCATGTCTCTTCTCATGGCTTCATCCTCATCG
Olea          ATCTTCTTTATAACTCTTTTTATTCTCAGCTTATTCAACATATTTCTTCACATGGGTTCATTGTTGTTG

cons          * ***** ** ***** ** **************    *** * ***** ***** *****  *  * *

Arabidopsis   CTCCTCAGTTATATAGTATCGCCGGACCAGACACAATGGATGAGATTAAATCAACGGCGGAGATTATGG
Olea          CACCCCAGTTATACAGCGTGGCAGGACCGGATGCAAATGAAGAAATCAAGATCACTGCTGATATAACGA

cons          * ** ******** **  * ** ***** **  ***  ** ** ** **    ** ** ** ** * *

Arabidopsis   ATTGGTTATCAGTAGGACTTAATCACTTTCTTCCAGCGCAAGTAACACCAAACCTATCCAAATTTGCCC
Olea          ACTGGTTATCAGAAGGACTGTGCCATTTTCTTCCAGCTAATGTTCGACCGGACTTGACAAAGCTAGGAC

cons          * ********** ******    ** ***********  * **   ***  ** *  * **  * *  *

Arabidopsis   TCTCCGGCCATAGCCGCGGTGGCAAAACCGCGTTTGCGGTCGCCTTAAAGAAATTTGGGTACTCCTCGA
Olea          TTGCTGGACACAGTCGAGGAGGAAAAGTCGCTTTCGCTCTAGCTCTAAGAAAGCAAGTC-A-----CTT

cons          *  * ** ** ** ** ** ** ***  *** ** **  * **  ***  **    *   *     *

Arabidopsis   ATCTAAAGATCTCGACATTGATCGGTATAGATCCAGTCGATGGAACAGGGAAAGGGAAACAAACCCCTC
Olea          CTTTGAAATTTTCAGCCGTGATAGGCATTGATCCTGTTGATGGAATGGAAAAGGGAAAACAAACACCTC

cons           * * **  * **  *  **** ** ** ***** ** *******  *  ** ** ******** ****

Arabidopsis   CTCCGGTGTTGGCTTACCTTCCAAACTCATTTGACCTAGACAAAACGCCTATACTTGTGATCGGTTCGG
Olea          CTCCGGTACTAAATTTTGTCCCCCATTCCTTCGATCTTGATAT---GGCAGTAATGGTTATAGGATCGG

cons          *******  *   **   * **  * ** ** ** ** ** *    * *  ** * ** ** ** ****
```

```
Arabidopsis    GGCTTGGTGAAACCGCTCGGAACCCATTATTCCCACCGTGTGCACCTCCCGGAGTGAATCACCGAGAGT
Olea           GATTGGGAGAAATGAAAAGGAATCCTCTTTTCCCTCCTTGTGCTCCTAAAGGAGTGAATCATCAAGACT

cons           *  * ** ****      **** **  * ***** ** ***** ***   *********** * *** *


Arabidopsis    TCTTTCGGGAATGTCAAGGTCCAGCATGGCATTTCGTTGCGAAGGATTATGGGCATTTGGACATGCTTG
Olea           TCTTTAACGAATGTCAAAAACCAGCTTTTTACTTTGTTGTGAAGGATTATGGACATCTTGATATGCTGG

cons           *****   *********   ***** *   * ** **** ************ *** * ** ***** *


Arabidopsis    ATGATGATACAAAAGGGATTAGAGGGAAGAGTTCTTATTGTTTGTGTAAGAATGGTGAAGAGAGGAGAC
Olea           ACGATGAGACAAAAGGGATTCGAGGGAAGACAACGCATTGTTTGTGCAAGAATGGGGAATCGAGGGAGC

cons           * ***** ************ *********   *  ********** ******** ***  ****   *


Arabidopsis    CAATGAGGAGATTCGTTGGTGGACTTGTTGTATCATTTTTGAAGGCTTATTTGGAAGGAGATGATCGTG
Olea           CGATGAGGAGATTTGTTGGGGGAATAGTGATTGCTTTCTTGAAAGCTTATTTGGAAGACAATTCAAGGG

cons           * *********** ***** *** * **  *  * ** ***** *************   **    * *


Arabidopsis    AATTAGTTAAGATCAAAGATGGGTGTCACGAGGATGTTCCCGTTGAAATTCAAGAGTTTGAGGTTATCA
Olea           AGTTGATGGCTATTCGAGATGGAAA---TGAGAAACTTCCTGTTGAGCTCCAAAACTCTGAATTTCTAA

cons           * **  *    **   ******       *** *  **** *****  * *** * * ***  ** * *


Arabidopsis    TGTAA
Olea           TTTAG

cons           * **
```

Figure S13*: OeCHL2* full-length coding sequence.

ATGAATTCTTCATCTACAACTCCCACCTTATCTTCTTCTTCCTCTAAAAATGCTTTTG
ATATTGGAAATCATTTAATTAAGCTGATTAAAATTGAGCCTAAAATAAGCAATAACA
GTGGTTCAGTCTGTTTTCCTCCGAAGCCACTTTTAATTGGGACACCAACAGAATCT
GGAGTTTTCCCAGTAGTTATACTCCTTCATGGTTATCTTCTTTATAACTCTTTTTATT
CTCAGCTTATTCAACATATTTCTTCACATGGGTTCATTGTTGTTGCACCCCAGTTAT
ACAGCGTGGCAGGACCGGATGCAAATGAAGAAATCAAGATCACTGCTGATATAAC
GAACTGGTTATCAGAAGGACTGTGCCATTTTCTTCCAGCTAATGTTCGACCGGACT
TGACAAAGCTAGGACTTGCTGGACACAGTCGAGGAGGAAAAGTCGCTTTCGCTCT
AGCTCTAAGAAAGCAAGTCACTTCTTTGAAATTTTCAGCCGTGATAGGCATTGATC
CTGTTGATGGAATGGAAAAGGGAAAACAAACACCTCCTCCGGTACTAAATTTTGTC
CCCCATTCCTTCGATCTTGATATGGCAGTAATGGTTATAGGATCGGGATTGGGAG
AAATGAAAAGGAATCCTCTTTTCCCTCCTTGTGCTCCTAAAGGAGTGAATCATCAA
GACTTCTTTAACGAATGTCAAAAACCAGCTTTTTACTTTGTTGTGAAGGATTATGGA
CATCTTGATATGCTGGACGATGAGACAAAAGGGATTCGAGGGAAGACAACGCATT
GTTTGTGCAAGAATGGGGAATCGAGGGAGCCGATGAGGAGATTTGTTGGGGGAA
TAGTGATTGCTTTCTTGAAAGCTTATTTGGAAGACAATTCAAGGGAGTTGATGGCT
ATTCGAGATGGAAATGAGAAACTTCCTGTTGAGCTCCAAAACTCTGAATTTCTAAT
TTAG

Figure S14: OeCHL2 full-length protein sequence.

M N S S S T T P T L S S S S S K N A F D I G N H L I K L I K I E P K I S N N S G S V
C F P P K P L L I G T P T E S G V F P V V I L L H G Y L L Y N S F Y S Q L I Q H I S S
H G F I V V A P Q L Y S V A G P D A N E E I K I T A D I T N W L S E G L C H F L P A
N V R P D L T K L G L A G H S R G G K V A F A L A L R K Q V T S L K F S A V I G I
D P V D G M E K G K Q T P P P V L N F V P H S F D L D M A V M V I G S G L G E M

K R N P L F P P C A P K G V N H Q D F F N E C Q K P A F Y F V V K D Y G H L D M L D D E T K G I R G K T T H C L C K N G E S R E P M R R F V G G I V I A F L K A Y L E D N S R E L M A I R D G N E K L P V E L Q N S E F L I

Figure S15. Amino acid alignment of predicted protein OeCHL2 with AtCHL2. Conserved residues are marked with an asterisk. Dots indicate residues with similar properties. Pink shades indicate conserved regions. Yellow and green shades and dashed lines indicate non-conserved or missing regions. The active site is highlighted with a red box.

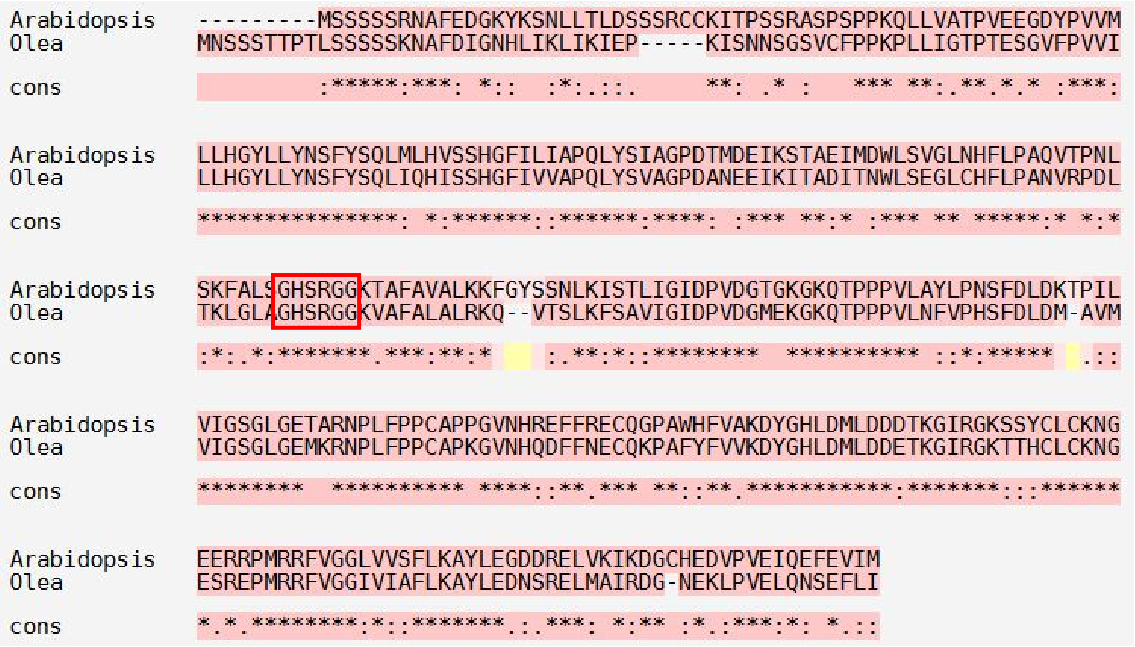

Figure S16: Common domain of the α/β-hydrolases superfamily protein OeCHL2

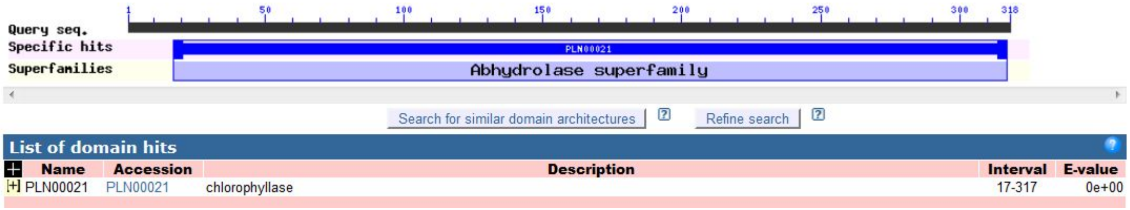

Figures S17-S24: The genome of *Olea europaea,* like Arabidopsis, encodes two SGR proteins, i. e., SGR1 and SGR2 (Kuai et al., 2018). The full-length coding sequence for *OeSGR1* consisted of 825 pb (Fig. S17), while the *OeSGR2* was 846 pb (Fig. S18). *OeSGR1* encoded a 274 protein with a calculated mass of 30.71 KDa (Fig. S19), sharing a similarity higher than 70% with AtSGR1 (Figure S20). The protein encoded by *OeSGR2* contained 281 amino acids and had a mass of 32.12 KDa (Fig. S21).

OeSGR2 has a similarity higher than 65% with AtSGR2 (Figure S22). Both proteins comprised the characteristic feature of the stay-green superfamily domain (Figure S23-S24) and the conserved cysteine-rich motif: C-X_3-C-X-C_2-F-P (Hörtensteiner, 2009). This terminal C-domain is essential for its function as it plays a critical role in the conformational change and self-interaction of the protein via the formation of inter- and intramolecular disulfide bonds (Xie et al., 2019).

Figure S17*: OeSGR1* full-length coding sequence

ATGGGCACTTTGAATGCAACTCCAGTGCTCCCATCAAACCTAAAATCTTCTTCACT
CAAACAAGAAAATAATTCTTTCTTGGTGTACAGAACAAGGAGAAGGAGAAGCAAGA
AGAACCACTCTATAGTCCCAGTGGCTAGATTATTTGGACCTGCAATATTTGAGGCA
TCAAAATTGAAGGTATTGTTCTTAGGAGTGGATGAGAAGAAGCATCCAGGGAAGC
TTCCAAGAACATATACACTTACACACAGTGATATAACCTCTAAGCTCACTCTTGCC
ATCTCTCAAACTATAAACAATTCCCAGTTACAAGGGTGGTACAATAGACTACAGAG
AGATGAAGTGGTGGCAGAGTGGAAGAAAATCAAAGAAAAAATGTCTCTTCATGTTC
ATTGTCACATAAGTGGTGGTCATTTCTTATTGGATCTCTGTGCTAGGCTCAGATAC
TACATCTTTTGCAAGGAACTTCCTGTGGTATTGAAGGCATTTGTTCATGGGGATGA
AAGCTTATTTGACAATCATCCAGACCTAAAGGAGGCATTGGTTTGGGTATATTTCC
ATTCCAACATACCAGAATTCAACAAGGTGGAGTGTTGGGGCCCACTCAAAGAGGC
ATCATCTCCTTCTAGTGGGGCCAATGGAGCCTACAACATTTGTGATGATGATAATC
CTCAGCCATGTGAGGAGGCTTGCAATTGTTGTTTCCCACCAATGGGTTTGATTCCA
TGGGCCCAAAATCTGTCTGGTGTGAGCGAGATTGGTGAATTCACCCCCAATGGCA
TGCACCAGGGGCAGCAGAATTTTGTCTCTGGTGGTGGTTCTTGA

Figure S18*: OeSGR2* full-length coding sequence

ATGGGCACTTTGACTGCTCCTCCTCTGGTGCTCCCATCAAACCTGAAGTCTAAAG
AGGAAAGTAATTCTTTCTTGGTGCAAGGAACAGGGAGAAGGAAAGGCAACAAAAC
CCAATCTTTACTCCCTGTGGCTAGATTATTTGGACCAGCAACATTTGAGGCATCAA
AATTGAAGGTATTGTTCTTAGGAGTTGATGAGAAGAAGCATCCAGGGAAGCTTCC
AAGAACATATACACTTACACACAGTGATATAACCTCTAAGCTCACTCTTGCCATCT
CTCAAACCATTAACAATTCCCAGTTACAAGGGTGGTACAACAGACTACAGAGAGAT
GAAGTGGTGGCAGAGTGGAAGAAAATCAAAGGAAAGATGTCTCTCCATGTTCATT
GTCACATAAGTGGTGGCCATTTCTTATTAGACCTCTGTGCTAGACTCAGATACTAC
ATCTTTTGCAAGGAACTCCCTGTGGTTTTGAAGGCATTTGTTCATGGGGATGAAAG
CTTATTTGACAACTATCCAGACCTACAAGAGGCTTTGGTTTGGGTATATTTCCATTC
CAACATACCAGAATTCAACAAGATGGAGTGCTGGGGTCCGCTCAAAGAAGCATCA
TCTCCATCCCGTAAGGTCAATGGGCCCCACAAATTATATGATGATCATAATCCCCA
GCCATGCAAGGAGGCTTGCAACTGTTGTTTCCCACCAATGAGTTTGATTACATGGT
CACAAAATGTAAATGGTGGGAGCACAATCAATGGCATGCACCAGAAGCATCAGAA
ATTTCTCTGGTGGTGGTTCTTTATATCTCTTTCATTTGGATTTAAAATTTTGCGTAAA
TGTAAATAG

Figure S19: OeSGR1 full-length protein sequence.

M G T L N A T P V L P S N L K S S S L K Q E N N S F L V Y R T R R R R S K K N H
S I V P V A R L F G P A I F E A S K L K V L F L G V D E K K H P G K L P R T Y T L T
H S D I T S K L T L A I S Q T I N N S Q L Q G W Y N R L Q R D E V V A E W K K I K
E K M S L H V H C H I S G G H F L L D L C A R L R Y Y I F C K E L P V V L K A F V
H G D E S L F D N H P D L K E A L V W V Y F H S N I P E F N K V E C W G P L K E
A S S P S S G A N G A Y N I C D D D N P Q P C E E A C N C C F P P M G L I P W A
Q N L S G V S E I G E F T P N G M H Q G Q Q N F V S G G G S

Figure S20: OeSGR2 full-length protein sequence.

M G T L T A P P L V L P S N L K S K E E S N S F L V Q G T G R R K G N K T Q S L L
P V A R L F G P A T F E A S K L K V L F L G V D E K K H P G K L P R T Y T L T H S
D I T S K L T L A I S Q T I N N S Q L Q G W Y N R L Q R D E V V A E W K K I K G K
M S L H V H C H I S G G H F L L D L C A R L R Y Y I F C K E L P V V L K A F V H G
D E S L F D N Y P D L Q E A L V W V Y F H S N I P E F N K M E C W G P L K E A S
S P S R K V N G P H K L Y D D H N P Q P C K E A C N C C F P P M S L I T W S Q N
V N G G S T I N G M H Q K H Q K F L W W W F F I S L S F G F K I L R K C K

Figure S21. Amino acid alignment of predicted protein OeSGR1 with AtSGR1. Conserved residues are marked with an asterisk. Dots indicate residues with similar properties. Pink shades indicate conserved regions. Yellow and green shades and dashed lines indicate non-conserved or missing regions. The active site (C-X_3-C-X-C_2-F-P) is highlighted with a red box.

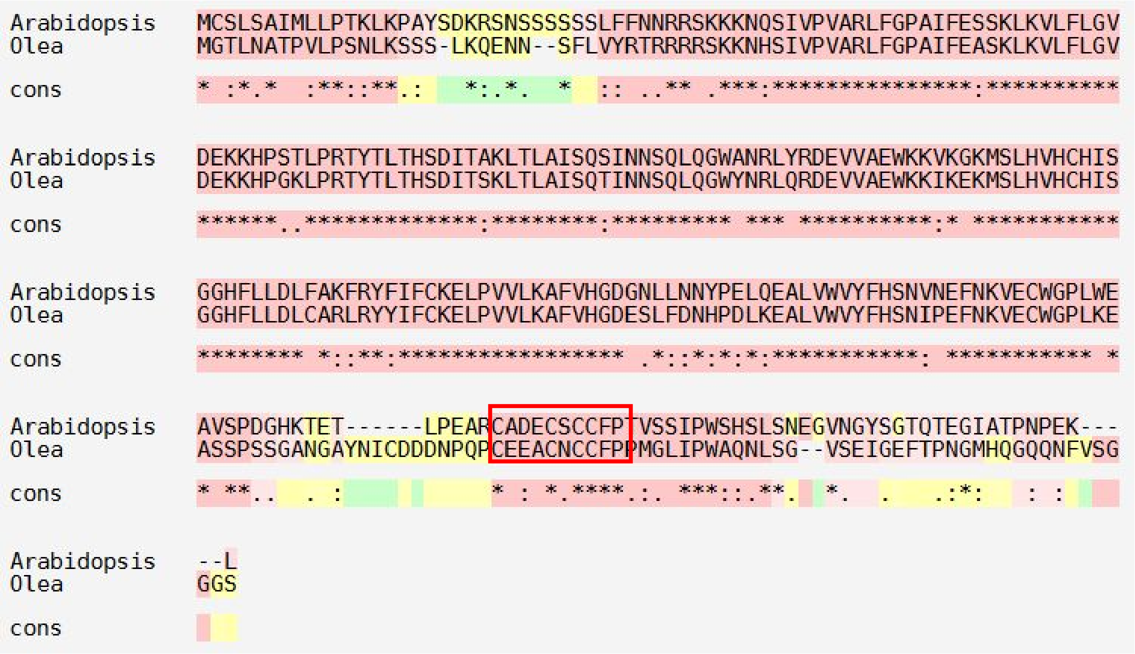

Figure S22. Amino acid alignment of predicted protein OeSGR2 with AtSGR2. Conserved residues are marked with an asterisk. Dots indicate residues with similar properties. Pink shades indicate conserved regions. Yellow and green shades and dashed lines indicate non-conserved or missing regions. The active site (C-X_3-C-X-C_2-F-P) is highlighted with a red box.

```
Arabidopsis    MCSLA-TNLLLPSKMKPVFPEKLSTSSLCVTT-RRSKMKNRSIVPVARLFGPAIFEASKLKVLFLGVDE
Olea           MGTLTAPPLVLPSNLKSKEE---SNSFLVQGTGRRKGNKTQSLLPVARLFGPATFEASKLKVLFLGVDE

cons           * :*: . *:***::*.      *.* *   * **.  *.:*::********* ***************

Arabidopsis    KKHPAKLPRTYTLTHSDITAKLTLAISQSINNSQLQGWANKLFRDEVVGEWKKVKGKMSLHVHCHISGG
Olea           KKHPGKLPRTYTLTHSDITSKLTLAISQTINNSQLQGWYNRLQRDEVVAEWKKIKGKMSLHVHCHISGG

cons           ****.**************:********:********* *:* *****.****:***************

Arabidopsis    HFFLNLIAKLRYYIFCKELPVVLEAFAHGDEYLLNNHPELQESPVWVYFHSNIPEYNKVECWGPLWEAM
Olea           HFLLDLCARLRYYIFCKELPVVLKAFVHGDESLFDNYPDLQEALVWVYFHSNIPEFNKMECWGPLKEAS

cons           **:*:* *:**************:**.**** *::*:*:***: ***********:**:****** **

Arabidopsis    SQHQHDGRTHKKSETLPELPCPDECKCCFPTVSTIPWSHRHYQHTAAD---ENVADGLLEIPNPGK---
Olea           SPSRKVNGPHKLYDDHNPQPCKEACNCCFPPMSLITWSQNVNGGSTINGMHQKHQKFLWWWFFISLSFG

cons           *  :: . .**  :     ** : *:****.:* *.**:.    :: :   ::  . *      .

Arabidopsis    -----SKG
Olea           FKILRKCK

cons                .
```

Figure S23: Common domain of the staygreen superfamily protein in OeSGR1

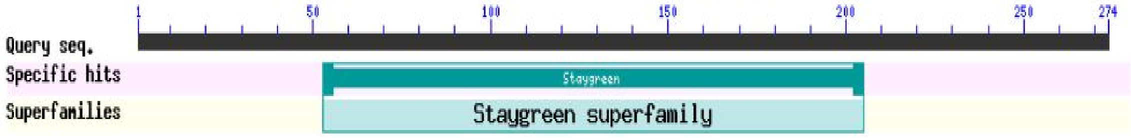

Figure S24: Common domain of the staygreen superfamily protein in OeSGR2

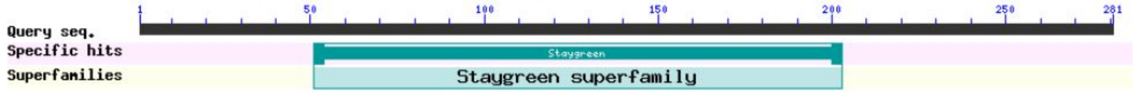

<u>Figures S25- S28</u>: The full-length coding sequence of *OePPH* contained 963 bp (Fig. S25) and encoded a protein of 320 amino acids (Figure S26) with a predicted mass of 35.89 KDa. The protein identity with AtPPH is 37%, but the overall protein sequence identity for PPH among different species has been described between 38% and 79% (Guyer et al., 2014). It is important to keep in mind that OePPH (Figure S27) is truncated, which can partially explain the identity results. Anyhow, PPHs belong to the α, β hydrolase superfamily, whose domain is present in OePPH (Fig. S28). In addition, the common PPH motif (VYXXGNSLGGXV, Figure 6, Guyer et al., 2014) is exhibited by OePPH, including the already identified active site Ser residue.

<u>Figure S25</u>: *OePPH* full-length coding sequence.

ATGGAAATTATATCCTGCCATTCTGCGCTCTGCTTTCATGTGGTAAATTTTGGTTAT
AGAGATGACATAAATTTGTGTCGTTCCAAGCTTCCCATTGTAAAAGGAAAATTGCC
ATTCCACGTTGTGGTTCGACCTGGTTGTGGATCTTCAAGATATTGTCATGCCAATA
TTTTAAGGTTAAAGAGATTAAACAGGTGTCAACCTTCTAGAACTGTGTGTTCACTAA
GCAGCGATGAAAATGTTAATTCAATCTTCTTAAGTGAAAGCTATAACTCTCACGTA
CTCGATGGGGAAGAGGATGTGATTAGTGTGATTGGTCGTGGCAAGTCAGTACCTA
AGGTCTTGATTCCTGGTTTGCCAGATGATTCAAATGGAGATTCTGTAGCTCCCATT
AGCAGTTGTTATTGGGAGTGGAAGCCTAAACTAAATGTCCATTATGAGAAATCGG
GATCTGAAAATCTAGATTCCCCACCAGTGCTTTTCCTTCCTGGATTTGGCGTGGGC
TCCTTTCATTATGAAAAGCAGCTAAAGGATCTTGGACGCGATTATAGAGCATGGGC
GCTTGATTTTCTGGGTCAGGGCATGTCCTTGCCATCTGAAGATCCAACTTTGCGG
CGTAAAGATGGAAACAAATCTATATTAGATGGAGAAAATCATGTTTGGGGTTTTGG
AGATGAAAGTGAAACTTGGGCAGAAGAACTTGTTTATTCAGTTGACTTGTGGAGG
GACCAAGTGCGCTATTTTGTAGAAGAGGTCATTAAGGAACCAGTTTATCTTGTGGG
AAATTCGCTTGGAGGATTTGTCGCACTCTATTTTGCAGCTTACCACCCTCAATTGG
TGAAGGGTGTAACCTTACTCAACTCCACGCCTTTCTGGGGGTTTCTCCCTAATCCC
TGCAAGATCTCCAAGATTATCGAGACTATTTCCATGGGCCGGAACATTTCCTCTTC
CTTCCAGTGTTAG

<u>Figure S26</u>: OePPH full-length protein sequence.

<u>M</u> E I I S C H S A L C F H V V N F G Y R D D I N L C R S K L P I V K G K L P F H V V
V R P G C G S S R Y C H A N I L R L K R L N R C Q P S R T V C S L S S D E N V N
S I F L S E S Y N S H V L D G E E D V I S V I G R G K S V P K V L I P G L P D D S N
G D S V A P I S S C Y W E W K P K L N V H Y E K S G S E N L D S P P V L F L P G
F G V G S F H Y E K Q L K D L G R D Y R A W A L D F L G Q G <u>M</u> S L P S E D P T L
R R K D G N K S I L D G E N H V W G F G D E S E T W A E E L V Y S V D L W R D
Q V R Y F V E E V I K E P V Y L V G N **S** L G G F V A L Y F A A Y H P Q L V K G V T
L L N S T P F W G F L P N P C K I S K I I E T I S <u>M</u> G R N I S S S F Q C

Figure S27: Amino acid alignment of predicted protein OePPH with AtPPH. Conserved residues are marked with an asterisk. Dots indicate residues with similar properties. Pink shades indicate conserved regions. Yellow and green shades and dashed lines indicate non-conserved or missing regions. The active site is highlighted with a red box.

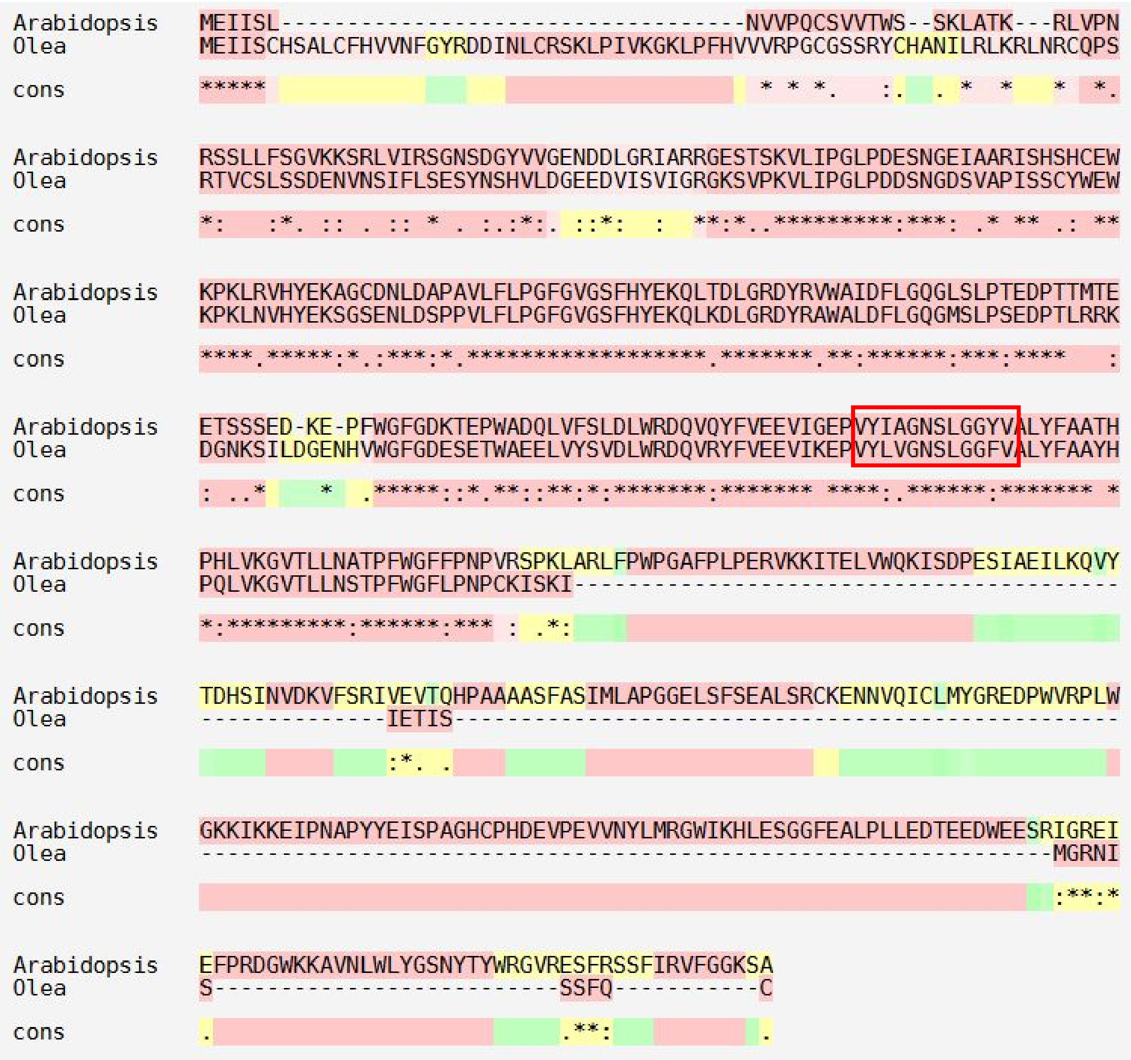

Figure S28: Common domain of the α/β-hydrolases superfamily protein OePPH

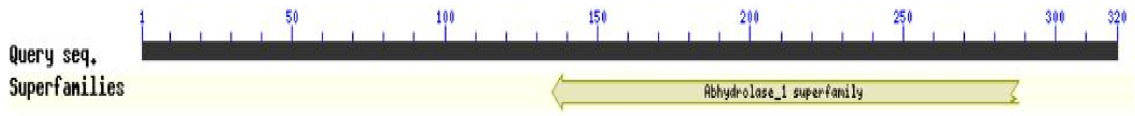

Figures S29-S32: The *OePAO* full-length coding sequence (Fig. S29) with 1632 bp encoded a 543 amino acid protein (Fig. S30) with a calculated mass of 61.36 KDa. OePAO shares a similarity with AtPAO of almost 80% and contains the domain of the Rieske-type iron-sulfur protein (Pruzinská et al., 2003, Fig. S31). In particular, OePAO displayed the Rieske centre, the mononuclear iron-binding site, and a proposed Ca+2-dependent protein kinase (CDPK) binding site (Figure 32) in agreement with the previous PPHs identified in other species (Chung et al., 2006).

Figure S29: *OePAO* full-length coding sequence.

ATGGCTGTTTCACTAGCATCAGGTACCCCTATATTCCCTTCGACTACCACTAGAAA
CAGTACAATCAACAACGCCATTGGCCCTCACTTCAATTGCAATCTTCCTTCGCAAA
AGAGAAGAAATTTCATCAACACTAGACTATGTGTTGCTACTCCACAAAGCACTCCT
ACTGCCAGCACTTCTGAAGAAAAACAGGATTCTGTATCGAGATTAGAAAATAATTT
TGAGGTTGAAATAGAGGATTCGGAAGAAAATTCATCAACAAAATTTTCTTGGAGAG
ACCATTGGTACCCTGTTTCGCTTGTAGAAGACATTGACCCACGTTATCCCACTCCT
TTTCAGCTACTGAATCGGGACCTGGTTCTGTGGTTTGATAGTACTGCTTCACAATG
GGTAGCTTTCGATGATAAATGCCCCCATCGCCTTGCACCTTTATCAGAGGGGAGG
ATAGATGAGAATGGACACTTGCAGTGTTCATACCACGGGTGGTCGTTTGATGGGT
GTGGGTCTTGTACTCGAATCCCACAGGCAGCATCAGAAGGGCCCGAGGCTCGTG
CTGTTAAGTCTCCGAGGGCATGTGCCACCAGGTTTCCAACGATGGTTTCCCAAGG
TCTTCTCTTTGTTTGGCCTGATGAGAATGGTTGGGAAAGAGCTCAAGCCACCAAA
CCCCCCATGCTGCCTGATGATTTTGATAAGCCCGATTTTTCATCTGTGACAATTCA
GCGCGATTTGTACTATGGCTATGATACTCTCATGGAGAATGTGTCTGATCCTTCCC
ACATTGACTTTGCACATCACAAGGTAACTGGGAGGAGAGATAGGGCAAAACCCTT
GCCATTCAAGATGGAAGGGACTGGACCATGGGGTTTTGCCGGTGCAAATGACGG
CAATCCAAAAATCAGTGCCAAGTTTGTTGCACCTTGCTACTATTTGAATAAAATTGA
GATCGACACAAAGCTCCCTCTCGTGGGTAATCAAAAATGGATAATATGGATATGTT
CCTTTAATGTTCCAATGGCACCTGGAAAGACACGCTCAATTGTTTGTAGTGCTCGA
AATTTCTTCCAGTTCACAATGCCAGGTCCTGCATGGTGGCAGGTGATTCCTCGCT
GGCATGAGCATTGGACTTCAAACAAGGTATATGATGGAGATATGATTGTACTTCAA
GGACAAGAAAAGATCTTTCTATCGAAGTCAAAGGATGGTTCTGGTGATGTCAACAA
ACAGTATACAAAGATCACCTTTACACCAACACAGGCCGATCGTTTTGTCCTGGCAT
TCAGGAATTGGCTGACGCGACATGGTAACAGCCAGCCTGAATGGTTTGGTACGG
CTGACCAACAACAGTTGCCATCTACTGTATTTTCCAAGCGCCAGATGTTGGATAGG
TATGAGCAGCACACGCTGAAATGTTCATCATGTAAGCAAGCTTTCACAACATTCCA
GACACTGCAGAAGTTCTTGATTGGAGCAGCTGTTATTTGCTCTGCAACAGCAGGG
ATTCCTCCAGACGTGCAAATACGGGTTATTTTGGGGGCAGTTGCAATTTTAAGCG
CAGGCCTAGCTTACTTTGCTTATGAAATCCAGAAGAACTTTGTGTTTATTGATTATG
TGCATGCTGATATTGATTGA

Figure S30: OePAO full-length protein sequence.

M A V S L A S G T P I F P S T T T R N S T I N N A I G P H F N C N L P S Q K R R N F
I N T R L C V A T P Q S T P T A S T S E E K Q D S V S R L E N N F E V E I E D S E E
N S S T K F S W R D H W Y P V S L V E D I D P R Y P T P F Q L L N R D L V L W F D
S T A S Q W V A F D D K C P H R L A P L S E G R I D E N G H L Q C S Y H G W S F
D G C G S C T R I P Q A A S E G P E A R A V K S P R A C A T R F P T M V S Q G L L
F V W P D E N G W E R A Q A T K P P M L P D D F D K P D F S S V T I Q R D L Y Y

G Y D T L M E N V S D P S H I D F A H H K V T G R R D R A K P L P F K M E G T G P
W G F A G A N D G N P K I S A K F V A P C Y Y L N K I E I D T K L P L V G N Q K W I
I W I C S F N V P M A P G K T R S I V C S A R N F F Q F T M P G P A W W Q V I P R
W H E H W T S N K V Y D G D M I V L Q G Q E K I F L S K S K D G S G D V N K Q Y
T K I T F T P T Q A D R F V L A F R N W L T R H G N S Q P E W F G T A D Q Q Q L P
S T V F S K R Q M L D R Y E Q H T L K C S S C K Q A F T T F Q T L Q K F L I G A A
V I C S A T A G I P P D V Q I R V I L G A V A I L S A G L A Y F A Y E I Q K N F V F I D
Y V H A D I D

Figure S31: Amino acid alignment of predicted protein OePAO with AtPAO. Conserved residues are marked with an asterisk. Dots indicate residues with similar properties. Pink shades indicate conserved regions. Yellow and green shades and dashed lines indicate non-conserved or missing regions. The red, green, and purple boxes represent the Rieske centre, the mononuclear iron-binding site, and a proposed Ca^{+2}-dependent protein kinase (CDPK) binding site (Chung et al., 2006)

```
Arabidopsis    MSVVLLSSTSA--TITKSQSKKIPFLSPTTKFPLKVSISPSRSKLFHNPLRVAAPPSVPTSDSTEEK--
Olea           MAVSLASGTPIFPSTTTRNSTINNAIGPHF--NCNLP-SQKRRNFINTRLCVATPQSTPTASTSEEKQD

cons           *:* * *.*.    : *. :*.    :.*      ::. * .* ::::.  * **:* *.**:.::***

Arabidopsis    ---RIEEE----YGGDKEEEGSEFKWRDHWYPVSLVEDLDPNVPTPFQLLGRDLVLWFDRNDQKWAAFD
Olea           SVSRLENNFEVEIEDSEENSSTKFSWRDHWYPVSLVEDIDPRYPTPFQLLNRDLVLWFDSTASQWVAFD

cons              *:*::      ..:*:..::*.*************:**. *******.********  . .:*.***

Arabidopsis    DLCPHRLAPLSEGRLDENGHLQCSYHGWSFGGCGSCTRIPQAATSGPEARAVKSPRACAIKFPTMVSQG
Olea           DKCPHRLAPLSEGRIDENGHLQCSYHGWSFDGCGSCTRIPQAASEGPEARAVKSPRACATRFPTMVSQG

cons           * ************:***************.************:.************** :********

Arabidopsis    LLFVWPDENGWDRANSIEPPRLPDDFDKPEFSTVTIQRDLFYGYDTLMENVSDPSHIDFAHHKVTGRRD
Olea           LLFVWPDENGWERAQATKPPMLPDDFDKPDFSSVTIQRDLYYGYDTLMENVSDPSHIDFAHHKVTGRRD

cons           ***********:**:: :** ********:**:*******:****************************

Arabidopsis    RAKPLPFKVESSGPWGFQGANDDSPRITAKFVAPCYSMNKIELDAKLPIVGNQKWVIWICSFNIPMAPG
Olea           RAKPLPFKMEGTGPWGFAGANDGNPKISAKFVAPCYYLNKIEIDTKLPLVGNQKWIIWICSFNVPMAPG

cons           ********:*..***** ****..*:*:******** :****:*:***:******:*******:*****

Arabidopsis    KTRSIVCSARNFFQFSVPGPAWWQVVPRWYEHWTSNLVYDGDMIVLQGQEKVFLAKSMESPDYDVNKQY
Olea           KTRSIVCSARNFFQFTMPGPAWWQVIPRWHEHWTSNKVYDGDMIVLQGQEKIFLSKSKDGS-GDVNKQY

cons           ***************::********:***:****** ***************:**:** :..  ******

Arabidopsis    TKLTFTPTQADRFVLAFRNWLRRHGKSQPEWFGSTPSNQPLPSTVLTKRQMLDRFDQHTQVCSSCKGAY
Olea           TKITFTPTQADRFVLAFRNWLTRHGNSQPEWFGT-ADQQQLPSTVFSKRQMLDRYEQHTLKCSSCKQAF

cons           **:****************** ***:*******: ..:* *****::*******::***  ***** *:

Arabidopsis    NSFQILKKFLVGATVFWAATAGVPSDVQIRLVLAGLSLISAASAYALHEQEKNFVFRDYVHSEIE
Olea           TTFQTLQKFLIGAAVICSATAGIPPDVQIRVILGAVAILSAGLAYFAYEIQKNFVFIDYVHADID

cons           .:** *:***:**:*: :****:*.*****::*..::::**. **   :* :***** ****::*:
```

Figure S32:

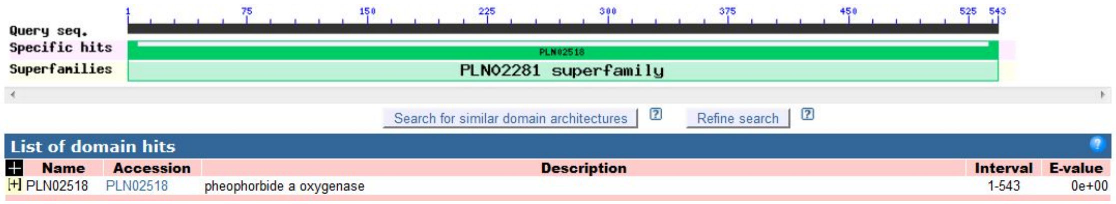

Figure S33: UV-Vis spectrum of an RCC formed during the *in vitro* PaO activity measurement in Arbequina fruits, showing the characteristics maxima (Jockusch & Kräutler, 2020).

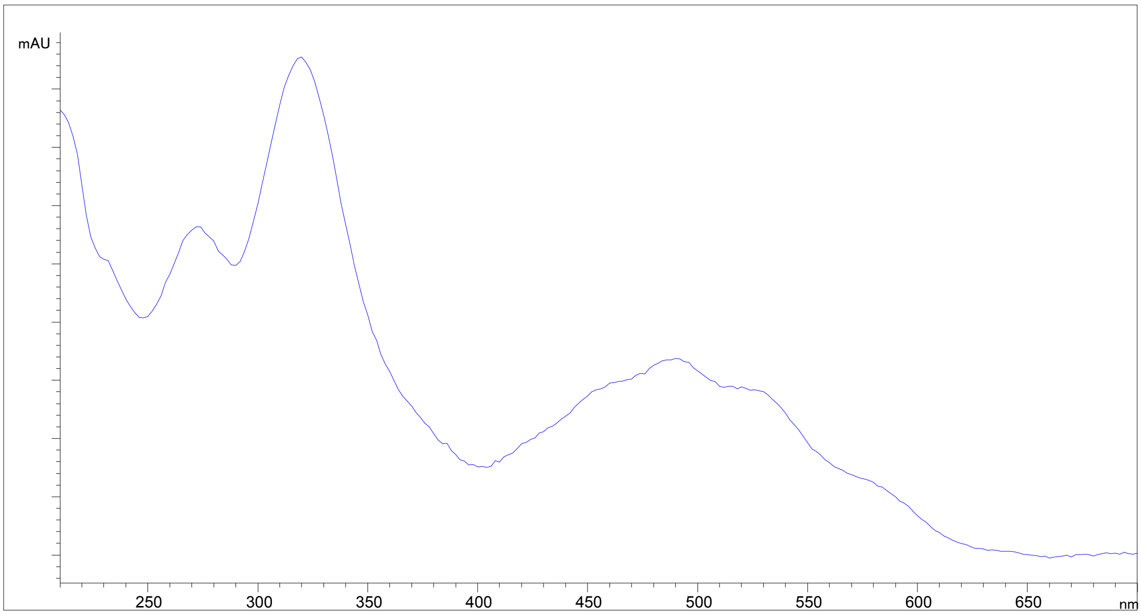

Supplement: Supplementary file 1 — jf2c00031_si_001.pdf [file jf2c00031_si_001.pdf]
